# Local‐Hybrid Functional With a Composite Local Mixing Function Built From a Neural Network and a Strong‐Correlation Model

**DOI:** 10.1002/jcc.70294

**Published:** 2026-01-24

**Authors:** Artur Wodyński, Martin Kaupp

**Affiliations:** ^1^ Institute of Chemistry, Theoretical Chemistry/Quantum Chemistry, Sekr. C7 Technische Universität Berlin Berlin Germany

**Keywords:** DFT, local hybrid functionals, neural‐network local mixing function, strong‐correlation factor, zero‐sum game

## Abstract

Due to their position‐dependent admixture of the exact‐exchange (EXX) energy density, local hybrid functionals (LHs) enable a flexible balance between reduced self‐interaction errors and smaller static‐correlation errors, allowing an escape from the usual zero‐sum game between these two central aspects of the development of density functional approximations. Recent LHs with strong‐correlation factors incorporated into their local mixing functions (LMFs) governing the position‐dependence of EXX admixtures have been particularly successful in this context. As only few exact constraints for LMFs are known regarding valence‐space behavior, some recent efforts have used machine learning in this context, and the recent LH24n functional with a “neural‐network LMF” (n‐LMF, DOI: 10.1021/acs.jctc.4c01503) has shown excellent performance for the large GMTKN55 test suite of main‐group energetics. However, so far the construction of n‐LMFs that also cover strong‐correlation effects has not been successful. Here we report the LH25nP functional that has an n‐LMF optimized in the presence of a fixed strong‐correlation factor. LH25nP‐D4 achieves a remarkable self‐consistent WTMAD‐2 value of 2.47 kcal/mol for the GMTKN55 set, the so far lowest value for a rung 4 functional. Mean absolute deviations of 2.4 kcal/mol for the large W4‐11RE reaction‐energy set are also the lowest known currently for rung 4. At the same time, very low fractional‐spin errors and excellent performance for the spin‐restricted dissociation of covalent bonds, as well as a curing of spin‐contamination problems in open‐shell transition‐metal complexes has been found, suggesting a clear deviation from the usual zero‐sum behavior. Transferability to organometallic transition‐metal energetics is so far less favorable, suggesting the need for a wider training of n‐LMFs that includes data for transition‐metal systems.

## Graphical abstract

A new local hybrid functional, LH25nP, is reported, that uses a neural‐network local mixing function for the position‐dependence of exact‐exchange admixture trained with a human‐designed strong‐correlation factor. It thereby escapes the usual zero‐sum game between delocalization and static‐correlation errors. LH25nP‐D4 gives the lowest WTMAD‐2 value for the GMTKN55 test suite so far for a rung 4 functional (2.47 kcal/mol), while improving by an order of magnitude on the proper spin‐restricted bond dissociation of covalent bonds.

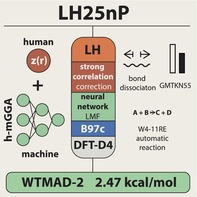

## 1 Introduction

Given the central importance of Kohn‐Sham density functional theory (KS‐DFT) in electronic structure theory, from quantum chemistry to solid‐state physics and material science, the development of new exchange‐correlation (XC) functionals, that is, of density functional approximations (DFAs), has continuously been a hot research topic for decades [1, 2, 3, 4, 5, 6, 7]. Apart from the treatment of dispersion interactions, for which good methods for many situations have been found, the central challenge of DFA development has been the zero‐sum game [7, 8, 9] between the minimization of self‐interaction errors (delocalization errors, sometimes denoted fractional‐charge errors, FCE) on one side of the coin [10, 11, 12, 13] and of static correlation errors (non‐dynamical correlation errors, sometimes labeled fractional‐spin errors, FSE) on the other side [14, 15]. Admixture of more exact exchange (EXX) of Hartree‐Fock‐like form in (global or range‐separated) hybrid functionals reduces self‐interaction errors but also increases static correlation errors as measured, for example, in the asymptotic energy error of spin‐restricted covalent bond dissociation curves [7, 8, 9].

Just as global hybrids are justified by the global adiabatic connection, local hybrids are grounded in a local adiabatic‐connection based on energy densities [16]. This yields a size‐consistent scheme that can be constructed free of one‐electron self‐interaction [17]. We have shown in recent years how the position‐dependent admixture of the EXX energy density in local hybrid functionals (LHs) and range‐separated local hybrid functionals (RSLHs) can provide the added flexibility needed to escape this zero‐sum game [7]. By contrast, most rung‐4 functionals (in global or range‐separated global form) lack the structure to overcome this trade‐off. Most recently RSLHs with correction terms for strong correlations as well as for delocalization errors in abnormal open‐shell regions, like ωLH23tdE [18] or ωLH25tdE [19], have been shown to provide (a) reduced static‐correlation errors, (b) small delocalization errors, and c) the correct asymptotic potential required to get good descriptions of charge transfer and related processes, while also being good functionals for general applications. For example, ωLH25tdE‐D4 has provided the so far lowest deviations (WTMAD‐2 value: 2.64 kcal/mol) [19] for the large GMTKN55 main‐group energetics data suite [20] of any rung 4 functional, indicative of top performance for mostly weakly correlated systems, while also reducing the errors in spin‐restricted bond dissociation by an order of magnitude compared to RSLHs without such correction terms. Other advantages of LHs and RSLHs over their global counterparts include excellent triplet excitation energies in TDDFT calculations or, for example, good NMR chemical shifts, and we point the reader to the pertinent reviews [6, 7] for these aspects. In terms of its input features, the DM21 “deep‐neural network” functional may also be considered an RSLH [21]. It achieves very small FCEs and FSEs, as it has been specifically trained on them, and it also provides reasonably good GMTKN55 performance (WTMAD‐2: 3.97 kcal/mol). However, due to its numerical complexity and probably suboptimal efficiency of the implementation, DM21 is currently not considered a functional useful for routine applications [22, 23]. We also mention the B13 [24] and KP16/B13 [25] coordinate‐space models for static correlation, which have inspired our most recent LHs and RSLHs, but which suffer from numerical problems when used in self‐consistent calculations.

The position dependence of EXX admixture in an LH or RSLH is governed by a so‐called local mixing function (LMF). Many forms of LMFs have been proposed over the years, a review up to 2019 is available [6]. Until recently, LMFs have been constructed heuristically from certain inhomogeneity functions, often used also in semi‐local functionals. The most widely used form, also by our group, has been the so‐called t‐LMF, a scaled ratio between von Weizsäcker and KS local kinetic energies [6, 26, 27]. While promising results have been obtained on this basis, including the abovementioned strong‐correlation corrected LHs and RSLHs, many deficiencies of such LMFs have been noted. For example, scaled t‐LMFs do not respect the high‐density and one‐orbital limits [28]. Moreover, using a new type of “x‐LMF” based on a ratio between semi‐local and exact exchange‐energy densities, we recently found that the shape of t‐LMFs and related forms seems to exacerbate the so‐called “gauge problem” arising from the ambiguity of exchange‐energy densities in the construction of LHs or RSLHs [29].

Indeed, apart from the abovementioned high‐density and one‐orbital limits [6], we know almost no further exact physical constraints, and none for the valence region, that would help us construct improved LMFs. A data‐driven approach to construct LMFs thus appears to be an attractive way forward. We have therefore recently constructed the first “n‐LMFs” as shallow neural networks, trained on relatively modest data sets of atomization energies and reaction barriers [30]. The initial LH24n‐B95‐D4 functional retained the B95c correlation functional [31] and other aspects from our earlier t‐LMF‐based LH20t‐D4 functional [32], and with DFT‐D4 dispersion corrections [33, 34, 35] it achieved a low WTMAD‐2 value of 3.49 kcal/mol [30]. Notably, this was obtained without addition of a so‐called calibration function (CF) used in other functionals like LH20t to suppress negative consequences of the gauge problem. Subsequent replacement of B95c by a more flexible and more widely optimized B97c‐type power‐series‐expansion correlation functional [36] provided LH24n‐D4, having a WTMAD‐2 value of 3.10 kcal/mol, again without use of a CF [30]. At the time this was the lowest value for a rung 4 functional (recently surpassed by the abovementioned RSLH ωLH25tdE‐D4 [19], see above). We also note the recent use of an n‐LMF to construct the first local double hybrid functionals [37]. However, neither the form of the n‐LMF nor the training data allowed for the incorporation of strong‐correlation effects, and so LH24n‐B95 and LH24n are still very much locked inside the zero‐sum game. A direct training of an n‐LMF on data including strong‐correlation input initially has not been very successful. We note that in strong‐correlation situations the overall LMF needs to be able to locally adopt negative values, and in human‐designed forms this local reduction of EXX admixture has been provided [7] by so‐called strong‐correlation factors $q_{\text{AC}}(\text{r})$ inspired originally by KP16/B13 methodology [25].

Here we pursue an intermediate approach to construct a new strong‐correlation‐corrected LH (scLH) by retaining a human‐designed $q_{\text{AC}}(\text{r})$ strong‐correlation function from a previous scLH, scLH23t‐mBR‐P [38], and then training an n‐LMF in the presence of such an sc‐factor. This gives the LH25nP functional, a well‐performing scLH with neural‐network LMF, the main result of the present work.

## 2 Theory

### 2.1 Strong‐Correlation Corrections to Local Hybrids: Padé Form Model

The general idea of coordinate‐space models of strong correlation along the lines of the B13 functional [24] is to extract the kinetic energy of static correlation from an interpolation along a local adiabatic connection. This has also been adhered to by the KP16/B13 model [25], with some differences in the interpolation and with a multiplicative $q_{\text{AC}}(\text{r})$ function. In our initial transfer of these ideas to scLHs [39, 40] we used a KP16/B13‐type multiplicative factor and a modified B13 version [41] of the exchange‐hole normalization ratios to detect in real space the regions of static correlation where enhancement is required, based on the relatively complicated and numerically demanding reverse Becke‐Roussel machinery underlying the B13, B05, and KP16/B13 models [24, 25, 42, 43].

In more recent models, for both scLHs [38] and their range‐separated variants (scRSLHs) [18, 19], we simplified our real‐space detection functions to depend just on the ratio z(r) between a semi‐local exchange‐energy density, $e_{x}^{\text{sl}}(r)$ (the modified Becke‐Roussel model [41] for the most successful variants so far, see Section S1.1 in the Supporting Information) and the exact exchange‐energy density, $e_{x}^{\text{ex}}(r)$, Equation (1). We have used different interpolation functions to construct $q_{\text{AC}}(\text{r})$ [38]. Here we will use the Padé form underlying the scLH23t‐mBR‐P functional [38] (used also for the ωLH23tdP scRSLH [18]), shown in Equation (2).

(1) $$z(r)=\max\left(\frac{e_{x}^{\text{sl}}(r)}{e_{x}^{\text{ex}}(r)}-1,0\right).$$

(2) $$q_{AC}(z(r))=0.5+d\cdot \frac{kz^{i}(r)}{1+kz^{i}(r)}.$$

This form had been chosen for its flexibility [38], when we made attempts to reduce the small unphysical maxima found in the spin‐restricted bond dissociation curves of main‐group diatomics with scLHs and scRSLHs (and with many other approaches to model such curves). d, k, and i are adjustable parameters optimized to reproduce accurate spin‐restricted bond dissociation curves, for scLH23t‐mBR‐P they were optimized for ${\text{N}}_{2}$ dissociation. This Padé form ensures a smooth behavior: for small values of z(r), the function is damped, while for larger values it asymptotically approaches d, thus avoiding unphysical overshooting (note that $q_{\text{AC}}(\text{r})$ should be between 0.5 for weakly correlated situations and somewhere below 1.0 in the most extreme strong‐correlation case).

As discussed above, the sc‐factor $q_{\text{AC}}(\text{r})$ becomes part of the LMF and reduces locally EXX admixture when the detection function z(r) becomes significant. We may write an LH in the form

(3) $$E_{\text{XC}}^{\text{LH}}=E_{\text{X}}^{\text{ex}}+\int (1-\text{LMF}(\text{r}))(e_{\text{X}}^{\text{sl}}(\text{r})-e_{\text{X}}^{\text{ex}}(\text{r})+G(\text{r}))\text{d}\text{r}+E^{\text{DC}},$$

where we may interpret the middle term as describing nondynamical correlation in analogy to the B05, B13, or KP16/B13 models. G(r) is the so‐called calibration function (CF) often used to deal with the gauge problem arising from the ambiguity of the exchange‐energy densities [6]. We will show further below that with the LMF constructed in this work, no CF is required, and we will therefore omit it. $E^{\text{DC}}$ denotes the semilocal dynamical‐correlation term of choice, typically B95c or B97c (see Section S1.4 in the Supporting Information for further details).

When integrating $q_{\text{AC}}(\text{r})$ into the LMF, the overall LMF of the scLH23t‐mBR‐P functional (which has a CF) becomes

(4) $$\text{tP‐LMF}(r)=1-2q_{\text{AC}}(r)\left[1-\text{t‐LMF}(r)\right],$$

where $\text{t‐LMF}(\text{r})=b\cdot \tau_{W}(\text{r})/\tau (\text{r})$ ($\tau_{W}(r)$ and τ(r) are the von Weizsäcker and KS kinetic energy densities, respectively, and b is an adjustable scaling factor). In the case of scLH23t‐mBR‐P, $q_{\text{AC}}(\text{r})$ also multiplies the B95c dynamical correlation contribution, as this was found empirically to produce overall better data [38]. Ideally, however, one would like to only use the local adiabatic connection to enhance the nondynamical correlation part, that is, the middle term of Equation (3). Indeed, the LH25nP functional reported here is the first successful attempt for an scLH where $q_{\text{AC}}(\text{r})$ acts only within the middle term of Equation (3).

### 2.2 The nP‐LMF Model: A Neural‐Network Local Mixing Function With Modulation by a Padé‐Form Strong‐Correlation Factor

The nP‐LMF underlying the new LH25nP functional model is given in Equation (5) in analogy to the tP‐LMF of Equation (4). That is, we replace the t‐LMF by an n‐LMF but retain the Padé‐form $q_{\text{AC}}(\text{r})$ from scLH23t‐mBR‐P, with unaltered parameters:

(5) $$\text{nP‐LMF}(r)=1-2q_{\text{AC}}(r)[1-\text{n‐LMF}(r)].$$

Note that the resulting LH25nP is an scLH without range separation. Introducing strong‐correlation effects into an scRSLH requires modifications to the range separation (one has to introduce long‐range contributions into the middle term, see Reference [18] for details). The combination of machine‐learned LMFs with scRSLHs is ongoing work in our lab. We also note in passing that RSLHs require about 2–3 times longer computations than LHs in current implementations based on semi‐numerical integration of the EXX energy densities [44].

In this construction, the fixed Padé‐form $q_{\text{AC}}(\text{r})$ encodes a large part of the strong‐correlation contributions, but we allow the n‐LMF to also make contributions by including fractional‐spin data in its training, which is done in the presence of $q_{\text{AC}}(\text{r})$ (see below). In this way the flexible neural‐network LMF training may correct possible deficiencies of the human‐designed $q_{\text{AC}}(\text{r})$ while also providing the required larger flexibility, for example compared to a t‐LMF, in the valence space for weakly‐correlated situations. We may also view the presence of $q_{\text{AC}}(\text{r})$ during n‐LMF training as a preconditioning that reduces parameter space and ensures better convergence. Similar uses of fixed function(al)s as preconditioners are known in other circumstances. An example is the use of the Fermi‐Amaldi potential to guide construction of smooth KS potentials with correct long‐range behavior from electron densities during the Zhao–Morrison–Parr procedure [45].

### 2.3 Neural‐Network Architecture

The n‐LMF is modeled by a multi‐layer perceptron (MLP) [46, 47] with three hidden layers of 128 neurons each and a single output neuron. Internally, the hidden layers use GELU [48] activation, while the output is passed through a scaled sigmoid function [49] mapping to [−1,1]. This matches the range of typical LMFs in strongly correlated regimes, even though $q_{\text{AC}}(\text{r})$ is already expected to lower the total LMF into the negative range in the most strongly correlated spatial regions even if the n‐LMF itself might still be positive. It is hoped that in this way the n‐LMF may, through appropriate training, improve upon possible deficiencies of the human‐designed $q_{\text{AC}}(\text{r})$ in regions of intermediately strong correlations. To aid faster and more stable training, we employ a logarithmic “squashing” transformation of the input features as used in the LH24n‐D4 functional [30]. Details of the neural‐network implementation are provided in the Supporting Information in Section S1.

The neural network is trained using post‐SCF data, with atomic‐orbital‐dependent features derived from orbitals computed self‐consistently with the scLH23t‐mBR‐P [38] functional. The feature set follows a hyper‐meta‐GGA design and includes nine components: spin‐resolved electron densities ($\rho_{\alpha }$, $\rho_{\beta }$), squared norms of their gradients ($\nabla \rho_{\alpha }\cdot \nabla \rho_{\alpha }$, $\nabla \rho_{\alpha }\cdot \nabla \rho_{\beta }$, $\nabla \rho_{\beta }\cdot \nabla \rho_{\beta }$), kinetic energy densities ($\tau_{\alpha }$, $\tau_{\beta }$), and exact‐exchange energy densities ($\varepsilon_{\text{ex}}^{\alpha }$, $\varepsilon_{\text{ex}}^{\beta }$). As in our previous work [30], the network is evaluated twice to preserve spin symmetry, once with the (α, β) ordering and once with (β, α), and the outputs are averaged to produce the final n‐LMF (see the Supporting Information).

For preliminary training of the nP‐LMF we retained the original parametrization of the other parts of exchange and correlation from LH24n‐D4, that is, PBE semi‐local exchange, a B97c [30, 36] power‐series expansion correlation functional, and DFT‐D4 [33, 34, 35] dispersion corrections, all with their previously optimized parameters [30]. The $q_{\text{AC}}(\text{r})$ parameters were taken from scLH23t‐mBR‐P.

### 2.4 Training Protocol and Datasets

The n‐LMF was trained against a composite loss function incorporating the W4‐17 [50] atomization‐energy set and the BH76 set [51, 52] of barrier heights. These two data sets were previously used during the training of n‐LMF of the LH24n‐D4 functional. To ensure larger chemical diversity, here we included additionally diet‐GMTKN55 [53] data, a 100‐reaction subset of the broader GMTKN55 [20] benchmark. Additionally, the extended version of the fractional‐spin FSE10 set [39] (see below) was used to include aspects of static correlation, based on artificial atomic systems with fractional‐spin occupations. diet‐GMTKN55 data were included to better pin down LMF behavior in covalent‐bonding regions. This provides a numerical stabilization of training compared to having W4‐17 and BH76 data only. Fixed D4 dispersion corrections from LH24n‐D4 were included during LMF‐training as diet‐GMTKN55 contains reactions sensitive to dispersion interactions. We did not remove overlap of diet‐GMTKN55 reactions with W4‐17 and BH76 data, as we assumed these overlaps to affect the training outcome to a negligible extent.

The FSE10 set consists of second‐ and third‐period p‐block atoms with fractional‐spin electron occupations changed, designed to capture static correlation. To illustrate the type of configurations included, consider the carbon atom with fractional‐spin occupations. A representative configuration can be expressed as:

(6) $$\begin{array}{lll} \left[core\right] & \; & {\left(2p_{x}\alpha \right)}^{0.5-\upsilon }{\left(2p_{y}\alpha \right)}^{0.5-\upsilon }{\left(2p_{z}\alpha \right)}^{0}\\ & \; & {\left(2p_{x}\beta \right)}^{0.5+\upsilon }{\left(2p_{y}\beta \right)}^{0.5+\upsilon }{\left(2p_{z}\beta \right)}^{0} \end{array}$$

We vary parameter υ from −0.5 to 0.0 in increments of 0.01, producing a smooth transition from a fully spin‐polarized to a spin‐depolarized configuration. Although the exact functional should return constant energy across this range, approximate functionals generally do not. The energy variation observed as υ changes provides a quantitative measure of the FSE and contributes to the overall loss function. Including FSE10 in the training of the LMF serves two purposes. First, it offers an opportunity to construct an LMF that further reduces FSE‐related errors in regions where the employed human‐designed $q_{\text{AC}}(\text{r})$ function may fail to detect strong correlations reliably, in particular regarding intermediate strengths. This is why the n‐LMF is allowed to cover the full range from −1 to 1. Second, we observed that without the inclusion of FSE10, the LMF tends to overfit other datasets at the expense of FSE performance, even in the presence of $q_{\text{AC}}(\text{r})$. To reinforce the association of negative LMF values specifically with strong correlation, we applied an additional penalty term (based on the mean square of negative LMF values) to the composite loss during training that discouraged negative LMF values on all training data except FSE10. This was meant to guide the neural network to learn that negative values of the LMF are permissible only in spatial regions characterized by appreciable static‐correlation errors.

For a balanced training we weighted the different test sets in the overall loss function as 0.5 (W4‐17), 1.0 (BH76), 0.1 (FSE10). The diet‐GMTKN55 data were weighted dynamically using the usual WTMAD‐2 formula [20] to account for varying reaction types. The overall loss is given by:

(7) $$\begin{array}{c} \begin{array}{ll} L_{\text{total}} & =0.5\;L_{\text{W4‐17}}+1.0\;L_{\text{BH76}}+0.1\;L_{\text{FSE10}}\\ & \;+\underset{r}{\sum }w_{r}^{\text{WTMAD‐2}}\;L_{r}+10^{3}\;L_{\text{neg‐LMF}},\; \end{array} \end{array}$$

where r∈diet‐GMTKN55. These choices of weights account for the larger variance of the FSE10 errors and for a need to avoid overrepresenting the W4‐17 atomization‐energy data compared to the BH76 barrier data. Note that this amounts to a global loss function based on energy differences. So far no attempts have been made to include local loss information from quantities at specific grid points [54], as no suitable high‐level reference energy or electron densities were available.

Training was performed in batches of single reactions. Each epoch thus contained 776 batches. That is, 776 updates of weights and biases were performed per epoch, with the neural network initially warmed up using a relatively high learning rate for about 500–700 epochs, after which the learning rate was gradually reduced and fine‐tuning was performed. We tested learning rates of $10^{-3}$, $10^{-4}$, and $10^{-5}$. Input features were updated twice during training using orbitals obtained from SCF calculations in Turbomole with intermediate versions of the functional. This reduced the gap between post‐SCF and SCF loss values and ensured consistency. We note in passing that fully self‐consistent training of an n‐LMF, while possible in principle, would substantially raise computational cost and strongly limit size and diversity of the training data. Such a treatment therefore is not common for ML‐based functionals. A small number of self‐consistent adjustments, as done also here, are typically used to narrow the post‐SCF vs SCF energy gap [55] or to regularize the density (see, e.g., Reference [56]). We find a few updates of the orbitals during an otherwise post‐SCF training a small price to pay to ensure consistency. The latter is confirmed by a small difference between the GMTKN55 WTMAD‐2 values for self‐consistent calculations with LH25nP‐D4 (2.47 kcal/mol) and the result of post‐SCF computations using the original scLH23t‐mBR‐P orbitals (2.63 kcal/mol).

### 2.5 Post‐Training Reparametrization

After training of the nP‐LMF in the way described above, the eight linear parameters of the B97c correlation functional and the parameters of the DFT‐D4 dispersion corrections were reparametrized against the full GMTKN55 database. In contrast to the LH24n‐D4 functional [30], no linear rescaling of the LMF was done during this refinement step. The linear $s_{8}$ scaling parameter of the D4 corrections was directly optimized with the linear B97c parameters. The non‐linear parameters $a_{1}$ and $a_{2}$ were optimized using a grid‐based strategy: multiple combinations of $a_{1}$ and $a_{2}$ within physically reasonable ranges were evaluated, and for each combination the remaining linear parameters were reoptimized.

We note in passing that the training does not cover core or asymptotic regions of the LMF, as these require typically self‐consistent or even TDDFT computations, which are not compatible with efficient post‐SCF training of a neural‐network LMF. We have shown previously for the LH23pt functional [28], how training of a core‐LMF contribution can be done, and we also showed that such a core‐LMF is largely independent of the valence part and therefore could also be added a posteriori. We furthermore note that B97c is a widely used meta‐GGA correlation functional that is free from one‐electron self‐interaction errors. It is not exact in the homogeneous electron‐gas limit but reasonably close. Of course, it does not include van‐der‐Waals interactions, which is why we added DFT‐D4 corrections. The wide use of B97c in many functionals, including very successful ones like ωB97M‐V [57], reflects its high flexibility, in particular regarding the separate description of same‐ and opposite‐spin channels.

## 3 Computational Details

The training and evaluation of the neural network‐based local mixing function (nP‐LMF) were carried out using a custom Python code based on the TensorFlow library [58]. Input features were obtained from a local version of Turbomole 7.8 [59, 60, 61] using the scLH23t‐mBR‐P [38] functional, def2‐QZVPPD [62] (for BH76, W4‐17 and FSE10) and def2‐QZVP [62] (for dietGMTKN55) basis sets [63], and Turbomole's “universal” auxiliary basis sets [64] for the RI‐J approximation [65, 66]. Grid sizes m3 or m4 were used to generate molecular orbitals used subsequently in feature extraction based on the spin‐resolved electron density, the square of the density gradient norm, the kinetic‐energy density, and the exact‐exchange energy density, using grid sizes 3 and 4 for BH76/W417/FSE10 and diet‐GMTKN55, respectively. Each grid point was processed through an element‐wise squashing function and a multi‐layer perceptron (MLP) with shared weights, consisting of three hidden layers (128 neurons each, GELU activation) and a single scaled sigmoid output mapping to [−1, 1]. This corresponds to ∼35k parameters inside the neural‐network part of the LMF, plus the fixed five parameters inside $q_{\text{AC}}(\text{r})$. See Section S1 in the Supporting Information for more details, and Table S1 for the parameters outside the neural network of LH25nP‐D4. The weights and biases of the neural network, as well as the corresponding reading algorithm, are provided as separate files (see Data Availability Statement).

After training, the neural network was embedded into a local development version of Turbomole to enable efficient, fully self‐consistent field (SCF) calculations with the trained nP‐LMF, including molecular gradients. Calculations on W4‐17 and BH76 were performed using def2‐QZVPPD [62] basis sets and grid size m3. For GMTKN55 [20], def2‐QZVP [62] basis sets and grid size m4 were employed. Diffuse functions were added to selected subsets in accordance with Reference [20].

Additional self‐consistent evaluations of LH25nP‐D4 were performed for real‐world transition‐metal organometallic reaction test sets, including the MOR41 [67] and ROST61 [68] reaction energies, and a modified subset (referred to as MOBH28 [69]) of the MOBH35 [70, 71, 72] set of reaction‐barrier heights. These calculations used def2‐QZVPP [62] basis sets together with def2‐ecp‐type Stuttgart‐Dresden scalar‐relativistic pseudopotentials [73] for 4d and 5d transition metals. In agreement with previous work [69], grid size m5 and the RI approximation were used consistently for all three test sets.

Optimizations of geometrical structures were carried out for the LMGB35 [74] and TMC32 [75] benchmark sets. We employed def2‐QZVPPD basis sets for LMGB35, and x2c‐TZVPall [76] basis sets together with the scalar‐relativistic X2C [77] method for TMC32. All optimizations were performed using analytical gradients implemented for local hybrid functionals [78], including range‐separated [44] variants. For the structure optimization of the mixed‐valent radical anions ${\text{V}}_{4}{{\text{O}}_{10}}^{.-}$ and ${\text{Al}}_{2}{\text{O}}_{4}^{.-}$ from the MVO10 benchmark [79], def2‐TZVP basis sets, gridsize 7 (or for comparison gridsize 3) as well as a Cartesian gradient convergence criterion of $10^{-4}$ a.u. were used (for consistency with earlier work [79], no D4 corrections were included).

Exact‐exchange energy densities were evaluated by semi‐numerical integration [80, 81, 82], more specifically by the semiJK algorithm [83], and Coulomb integrals used the RI‐J approach with “universal” auxiliary basis sets. All SCF calculations were converged to $10^{-7}$ Hartree. We observed no significant SCF convergence issues across the training and test systems, confirming the numerical stability of the functional with the learned nP‐LMF.

## 4 Results

### 4.1 GMTKN55 Benchmark Evaluations

**FIGURE 1:**
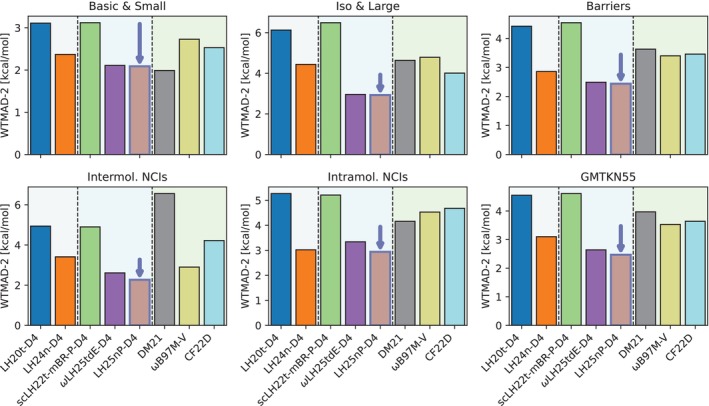
Comparison of WTMAD‐2 values across five GMTKN55 subcategories and overall dataset performance for various LH, RSLH, and other rung 4 functionals trained on large datasets (DM21 [21], ωB97M‐V [57, 84], CF22D [85]).

Figure 1 summarizes the WTMAD‐2 values (in kcal/mol) of LH25nP‐D4 together with a representative set of modern hybrid and local‐hybrid DFAs for the full GMTKN55 benchmark. WTMAD‐2 values for the usual five subcategories, Basic & Small, Iso & Large, Barriers, Intermolecular NCIs, and Intramolecular NCIs, are also provided.

LH25nP‐D4 achieves the lowest overall WTMAD‐2 value (2.47 kcal/mol) among all functionals on rung 4 of the usual ladder hierarchy of DFAs [86], which includes global hybrids, range‐separated hybrids, LHs, RSLHs, and so on. This value is already clearly in the range of many rung 5 functionals [87, 88] (only a few recent double hybrids achieve lower values, see References [37, 89, 90], and references therein) but is achieved without use of information on the virtual orbital space. The largest relative improvement for LH25nP‐D4 compared to our earlier LHs is observed for non‐covalent interactions (NCIs): the WTMAD‐2 value for intermolecular NCIs of 2.27 kcal/mol is more than 0.6 kcal/mol lower than that of the next‐best full‐range LH (LH24n‐D4) and slightly better than that of the best RSLH, ωLH25tdE‐D4 (2.61 kcal/mol). Similar improvements are seen in the Iso & Large category, which also has significant contributions from dispersion interactions. That is, improvements due to the nP‐LMF compared to earlier LHs are clearly seen in these data, which may be considered to cover largely systems without strong correlations. Improvements compared to LH24n‐D4 are nevertheless to some extent due to the inclusion of the strong‐correlation factor. Improvements compared to earlier LHs like LH20t‐D4 or its scLH variant scLH23t‐mBR‐P‐D4 reflect not only the more flexible LMF but also use of the more flexible B97c correlation functional, which helps improve NCIs in combination with the DFT‐D4 terms (see References [19, 30] for further analyses of this aspect). The improved intermolecular NCIs may also partly result from the inclusion of diet‐GMTKN55 data in the optimization procedure. Our comparisons between LHs and RSLHs and the performance of the less parameterized ωLH25tdE‐D4 so far point to the potential of further improvements on GMTKN55 energetics when going to a corresponding RSLH, which is ongoing work. In any case, an LH with top performance in this area is useful in its own right, given the somewhat lower computational requirements for an LH compared to an RSLH [44] (see Theory section). We also emphasize the fact that LH25nP‐D4 achieves its performance, including that for NCIs, without using a CF to deal with the gauge ambiguity of exchange‐energy densities. That is, like the n‐LMF of LH24n the nP‐LMF of LH25nP helps suppress the spurious nondynamical correlation contributions that render intermolecular interaction potentials too repulsive for, e.g., LHs and RSLHs based on a t‐LMF when used without a CF (see also Reference [29]). See below for further analyses.

Compared to other prominent rung 4 functionals, LH25nP‐D4 improves not only in the NCI and Iso & Large categories but also on reaction barriers. The barrier WTMAD‐2 value is, for example, almost one kcal/mol lower than that of the ωB97M‐V RSH and CF22D GH and even more compared to DM21, which may be considered a highly parameterized RSLH with essentially black‐box character. DM21 is particularly good in the Basic & Small category, where it still slightly outperforms LH25nP‐D4. We note in passing that the recent deep‐neural‐network functional Skala [55] achieves a WTMAD‐2 value of ca. 3.89 kcal/mol, which is about 1.4 kcal/mol higher than that of LH25nP‐D4 but still impressive given that the computational cost of Skala apparently is closer to that of a rung 3 functional.

The largest MAD values with LH25nP‐D4 for individual subsets of GMTKN55 (see Table S2 in the Supporting Information) are found for the MB16‐43 (“mindless benchmarking”) subset, the C60ISO subset of fullerene isomerization reactions, and the DIPCS10 subset of double ionization potentials [20, 91]. Weaker performance for the double ionization potentials may reflect that LH25nP‐D4 is a full‐range LH without long‐range corrections and therefore does not exhibit the correct asymptotic potential. We are currently re‐evaluating the C60ISO reference data and see evidence that improved reference data will shift the performances of different types of DFAs significantly (M. Reimann, M. Kaupp, in preparation). We therefore view this result with some caution. Note that the weight of C60ISO within GMTKN55 is small. The MAD of 20.2 kcal/mol for MB16‐43 is appreciable but comparable to those of many other rung 4 or rung 5 functionals.

Given that the nP‐LMF is a highly parameterized object, and we have used the diet‐GMTKN55 set, as well as W4‐17 and BH76, as part of the training data, possible overtraining is a concern. We have evaluated this for LH25nP‐D4, LH24n‐D4, and ωLH25tdE‐D4 in a comparative way by assessing their performance for a reduced “non‐diet‐GMTKN53” set obtained by removing the 100 reactions of diet‐GMTKN55 as well as the W4‐11 [92] (which strongly overlaps with W4‐17) and BH76 data from GMTKN55. Results are shown in Table 1. Focusing on the overall WTMAD‐2 value for this reduced set, we get similar performance for LH25nP‐D4 and ωLH25tdE‐D4. This should be compared to the 2.47 and 2.62 kcal/mol of these two functionals for the full GMTKN55 set. As ωLH25tdE is a significantly less parameterized functional than LH25nP, we may take the somewhat larger increase for the latter upon what is essentially a removal of the training data from evaluation as an indication of the magnitude of overtraining. The data would suggest that we see an overtraining by ca. 0.15 kcal/mol. For LH24n‐D4 the increase from the full GMTKN55 value of 3.10 kcal/mol is more similar to that for ωLH25tdE‐D4. This suggests even less overtraining of the n‐LMF in that case, where only W4‐17 and BH76 had been used for LMF‐training. Overtraining of LH25nP‐D4 could be suspected from its WTMAD‐2 of only ca. 1 kcal/mol for the smaller diet‐GMTKN55 set used in the training. However, the above results for the largely orthogonal “non‐diet‐GMTKN53” indicate that such overtraining is a comparably small issue. Transferability of highly trained functionals is a separate aspect that will be addressed further below.

**TABLE 1:** Comparison of selected dispersion‐corrected functionals for the total WTMAD‐2 value and subcategory values of “non‐diet‐GMTKN53” in kcal/mol (see text).

	Basic & Small	Iso & Large	Barriers	Intermol. NCIs	Intramol. NCIs	Full
LH25nP‐D4	2.90	3.08	2.59	2.37	3.06	2.82
ωLH25tdE‐D4	2.66	2.98	2.20	2.63	3.38	2.83
LH24n‐D4	2.97	4.40	2.39	3.45	3.06	3.32

### 4.2 Evaluation for the Large, Automatically Generated W4‐11RE Reaction‐Energy Set

While the overall performance on GMTKN55 is heavily influenced by non‐covalent interactions, it is equally important to assess the accuracy for standard reaction energies. W4‐11RE [93] is a set of more than 11,000 highly accurate reaction energies derived without extra quantum‐chemical computations from the W4‐11 atomization‐energy set of only 140 molecules. It has been argued that performance on atomization energies does not always translate into accurate reaction energies, making this test set a valuable tool for further validation.

We therefore evaluated the LH25nP‐D4 functional on W4‐11RE, using the same settings as applied in our previous work [19]. Table 2 shows that LH25nP‐D4 achieves an MAD of 2.40 kcal/mol, improving upon the ωLH25tdE‐D4 scRSLH, which reaches 2.65 kcal/mol. This 0.25 kcal/mol reduction even in the absence of range separation highlights the advantages of the flexible nP‐LMF. To the best of our knowledge, LH25nP‐D4 gives the best performance of a rung 4 functional so far also for this test set. Only a few rung 5 functionals have been reported to give even lower errors [93, 94, 95]. The evaluations in References [94] and [95] are not comparable, as energies involving the ${\text{C}}_{2}$ and ClOO molecules were missing due to convergence problems, and ${\text{C}}_{2}$ is known to produce very large errors for functionals without an adequate treatment of strong correlations (cf., e.g., Table S3 in the Supporting Information for errors in ${\text{C}}_{2}$ atomization energies). The best rung 5 functional in Reference [93] was PWPB95, giving an MAD of 2.36 kcal/mol, just slightly below that of LH25nP‐D4.

**TABLE 2:** Performance of selected rung 4 functionals for the mean absolute deviation (MAD) of the W4‐11RE reaction‐energy test set.

Functional	MAD (kcal/mol)
LH25nP‐D4	2.40^a^
ωLH25tdE‐D4	2.65^b^
LH24n‐D4	3.10^b^
ωB97M‐V	3.25^b^
LH20t‐D4	3.63^b^
M05‐2X	4.68^b^

^a^ This work.

^b^ Reference [19].

### 4.3 Performance for Strong‐Correlation Cases and Spatial Shapes of the nP‐LMF

**FIGURE 2:**
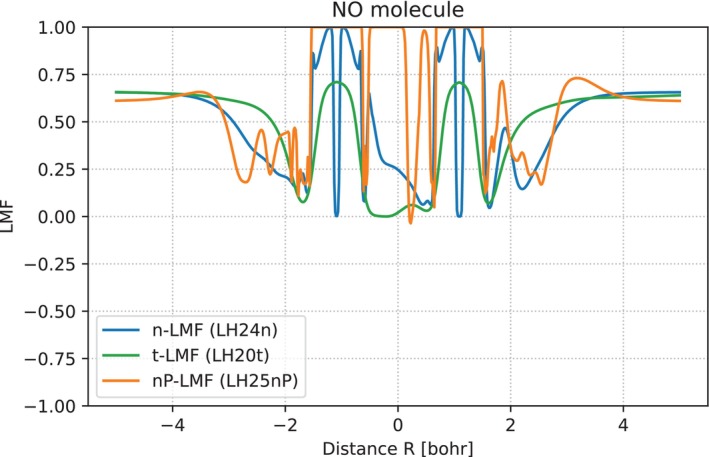
LMF‐shapes along the bond axis for the NO molecule, comparing the nP‐LMF of LH25nP‐D4 (orange), the n‐LMF of LH24n‐B95‐D4 and LH24n‐D4 (blue), and the t‐LMF of LH20t‐D4 (green). See Reference [6] and work cited therein for detailed discussions of the different spatial regions contributing to LMF‐shapes.

Compared to black‐box deep‐neural‐network functionals like DM21 [21] or Skala [55], our more limited application of machine learning to just the LMF of an otherwise well‐defined LH has the advantage that we retain much more insight into the inner workings of our functional. The LMF is a real‐space quantity that we may plot on a grid for a given molecule, allowing us to link the spatial form of the LMF to some extent to the performance of the associated functional for different quantities. That is, we may compare the position‐dependence of EXX admixture in LH25nP‐D4 to those in other LHs, including those with human‐designed LMFs. For example, Figure 2 shows LMF shapes along the bond axis for the NO molecule at its equilibrium structure, comparing the nP‐LMF of LH25nP‐D4, the n‐LMF of LH24n‐D4, as well as the t‐LMF of LH20t. Additional comparisons for other systems are provided in the Supporting Information (Figures S1, S2, and S6).

In general, the nP‐LMF shows a bit more wiggles than the previous n‐LMF of LH24n‐D4 [30], which might indicate some overparameterization. However, we did not observe any SCF convergence issues that could be attributed to these oscillations. The nP‐LMF goes to 1.0 not only in a relatively large region around the nuclei but also in part of the bonding region, where the t‐LMF goes to zero by construction and the previous n‐LMF has significant values but still rather far from 1.0. Similar to earlier findings for the n‐LMF [30], the asymptote of the nP‐LMF far from the nuclei appears to go towards values around 0.5–0.7, which is similar to the 0.715 of the scaled t‐LMF of LH20t‐D4. We note that neither this asymptotic region nor the core region are in any way covered by the training data, so it remains unclear if there is any meaning to those asymptotic values. This is why in this work we will focus only on quantities related to the valence behavior. Large EXX admixtures in the bonding region appear to help with the suppression of gauge artefacts without need for a CF [29, 30]. All three LMFs exhibit low values just outside the core region, the nP‐ and n‐LMFs also have narrow peaks following these areas both outside and inside the bonding region.

Locally negative regions for hydrogen‐containing molecules at equilibrium structure like HCl and HS (Figure S1) outside the hydrogen atoms likely do not reflect strong correlations but might be due to self‐interaction errors in the semi‐local exchange‐energy density entering the $q_{\text{AC}}(\text{r})$ function via the detection function z(r). We have discussed this previously in the context of artefacts found for the electron affinity of the hydrogen atom computed with scRSLHs from the highest molecular orbital energy of the hydride anion [18]. This may have to be addressed in future work by improved damping of $q_{\text{AC}}(\text{r})$ or z(r) at low values, or by improving the real‐space detection function z(r) in other ways.

As we start stretching covalent bonds moderately, for example, for ${\text{N}}_{2}$ (Figure S2) or NO (Figure S3), the high nP‐LMF values in the bonding region at the equilibrium structures decrease with bond elongation. This may be interpreted as an indication that the model captures the emergence of static correlation upon moderate bond stretching even before the $q_{\text{AC}}(\text{r})$ function starts to deviate significantly from 0.5 (it does not for those moderately stretched bonds). We speculate that the neural‐network part of the LMF may have learned some aspects of moderate static correlation from the training data. Deeper exploration of this aspect will require further work.

**FIGURE 3:**
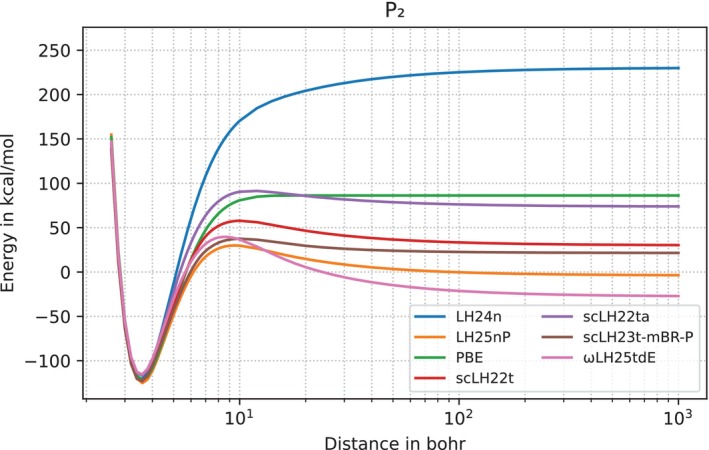
Dissociation curve for the ${\text{P}}_{2}$ molecule using spin‐restricted KS calculations. Results for LH25nP are compared to scLH23t‐mBR‐P (with the same $q_{\text{AC}}(\text{r})$ function), related LHs, scLHs, and scRSLHs (scLH22t, scLH22ta, ωLH25tdE, LH24n), as well as PBE.

**FIGURE 4:**
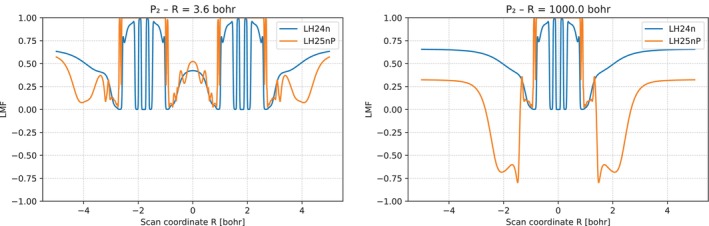
Comparisons of the nP‐LMF of LH25nP‐D4 and the n‐LMF of LH24n‐D4 and LH24n‐B95‐D4 for the ${\text{P}}_{2}$ molecule along the bond axis at equilibrium structure (at 3.6 bohr) and near dissociation (at 1000 bohr).

As we stretch bonds further, the $q_{\text{AC}}(\text{r})$ function starts to rise significantly above 0.5 and thereby diminishes EXX admixture locally, even to the point where it can become negative. By this mechanism scLHs improve on spin‐restricted bond dissociation curves [38, 39, 40]. We show this for the ${\text{P}}_{2}$ molecule in Figure 3, with LMF plots at equilibrium distance and at 1000 bohr in Figure 4. The dissociation curve of LH25nP agrees with those of other functionals near equilibrium, exhibits a relatively small unphysical maximum (“hump”) at intermediate distance and converges to an energy close to the sum of the two separate, fully spin‐polarized atoms. The value at dissociation is the best of the functionals shown in Figure 3 and in fact the best of any of our scLH or scRSLH functionals so far (see below for a wider evaluation). LH24n and PBE have been included as functionals without explicit strong‐correlation corrections. They converge to far too high energies at dissociation, due to static‐correlation errors (twice the fractional spin error of the P atom, see below). The fact that the error of LH24n is much larger than that of PBE at the asymptote reflects the zero‐sum game [7, 8, 9], where larger EXX admixture helps reduce delocalization errors but increases static‐correlation errors. The intermediate maximum of LH25nP is similar to those of other scLHs or scRSLHs and in fact similar to many other functionals that include some corrections for static correlation, such as DM21 [21] or even RPA‐based functionals on rung 5 [96]. We show spin‐restricted bond dissociation curves for other main‐group diatomics in Figures S4, S5 in the Supporting Information.

The LMFs for ${\text{P}}_{2}$ in Figure 4 show that the corrected nP‐LMF and the uncorrected n‐LMF behave similarly in the valence region (apart from aspects discussed above) at the equilibrium structure, while the effects of $q_{\text{AC}}(\text{r})$ for the nP‐LMF become very large near dissociation, with significantly negative areas left and right of each of the phosphorus atoms (only one of them is inside the plot at this large distance).

We often use deviations of the spin‐restricted curves at very large distances from the sum of the energies of the spin‐polarized atoms for ten second‐ and third‐period main‐group diatomics to evaluate static‐correlation errors (DISS10 test set) [40]. We also evaluate the height of the unphysical maxima compared to the energy at 1000 bohr for the same set of molecules as a measure of intermediate FSE situations (see below) as the HUMP10 set [40]. Table 3 compares data for these two sets with LH25nP and the other functionals evaluated also in Figure 3. LH25nP provides the lowest DISS10 MAE of all functionals in the table, including ωLH25tdE and scLH23t‐mBR‐P. The latter has the same $q_{\text{AC}}(\text{r})$ function as LH25nP. The lower DISS10 value for LH25nP suggests that the LMF training may have recovered some additional strong‐correlation effects beyond what is encoded in $q_{\text{AC}}(\text{r})$. We note in passing that DM21 exhibits almost vanishing MAE for DISS10 [40] (1.2 kcal/mol). Possibly the less flexible form and training of LH25nP may still sacrifice some performance for such strong‐correlation data as it attempts to improve upon more weakly correlated situations (recall that LH25nP‐D4 gives a GMTKN55 WTMAD‐2 value of 2.47 kcal/mol compared to a value of 3.97 kcal/mol for DM21 [21]).

**TABLE 3:** Mean absolute and signed errors (MAE, MSE) in kcal/mol for the DISS10 and HUMP10 sets with selected functionals.

		MAE	MSE
LH24n	DISS10	157.4	157.4
LH25nP	DISS10	10.2	3.9
	HUMP10	27.1 (31.0^a^)	—
PBE	DISS10	61.2	61.2
scLH22t	DISS10	27.8	23.3
	HUMP10	26.6 (50.0^a^)	—
scLH22ta	DISS10	54.2	54.2
	HUMP10	14.8 (69.0^a^)	—
scLH23t‐mBR‐P	DISS10	21.0	20.3
	HUMP10	13.1 (33.4^a^)	—
ωLH25tdE	DISS10	36.8	−3.1
	HUMP10	48.4 (45.4^a^)	—

*Note:* The MSE is not reported for HUMP10, as its definition always yields positive values by construction. As the uncorrected functionals LH24n and PBE do not exhibit maxima, no HUMP10 values are given for them. Calculations without dispersion corrections.

^a^ Values in parentheses give the “HUMP10” MAEs when comparing to the correct asymptotic energy of two spin‐polarized atoms rather than to the spin‐restricted value at 1000 bohr for the given functional.

Interestingly, for HUMP10 LH25nP performs comparably to DM21, where we found a value of ca. 37 kcal/mol [40] (in Reference [21] the unphysical maximum could only be removed by employing fractional occupations). The scLH23t‐mBR‐P model and scLH22ta with an “undamped” $q_{\text{AC}}(\text{r})$ give the lowest HUMP10 values. LH25nP gives a value comparable to other scLHs and scRSLHs. These “barrier” values have to be taken with some caution, as they are given relative to the energy at 1000 bohr, which may deviate to different extents from the exact asymptote. Values in parentheses in Table 3 therefore provide barrier values relative to the correct asymptotic energy. Here LH25nP is now among the best‐performing functionals, similar to scLH23t‐mBR‐P. In any case these small barriers at intermediate distances reflect to some extent subtle aspects of simulating intermediately strong correlations. We note in passing that the DISS10/HUMP10 MAEs of LH25nP are below those of the KP16/B13 functional, 19.2/53.4 kcal/mol, and slightly below those of B13, 13.6/35.8 kcal/mol [40].

**FIGURE 5:**
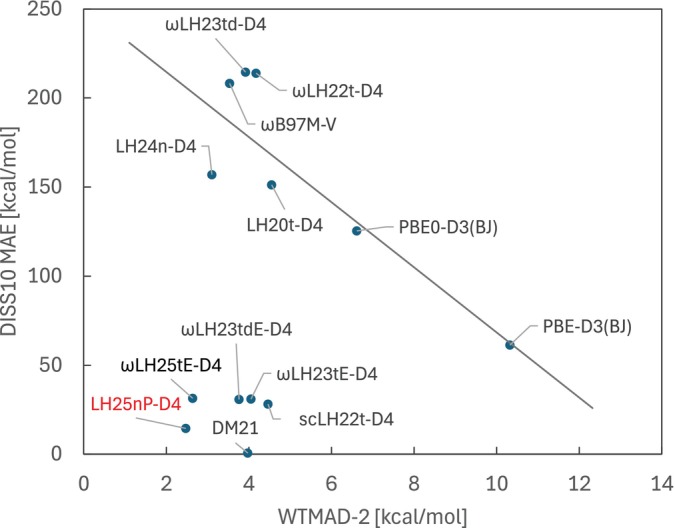
Indication of the usual zero‐sum game and the escape from it by strong‐correlation‐corrected functionals as indicated by plots of the DISS10 and GMTKN55 WTMAD‐2 performance for different functionals.

The simultaneously good description of strong‐correlation situations, as exemplified by the DISS10 data, and of more weakly correlated situations as represented by the GMTKN55 WTMAD‐2 values is a clear indication that LH25nP escapes to a significant extent the usual zero‐sum game between these two aspects. This is shown by plotting these two quantities against each other [7], as in Figure 5. Most functionals fall roughly on a line with negative slope that exemplifies the zero‐sum behavior, including LH24n‐D4. LH25nP‐D4 is significantly shifted to the lower left part of the plot, as are a number of other functionals with strong‐correlation terms.

**FIGURE 6:**
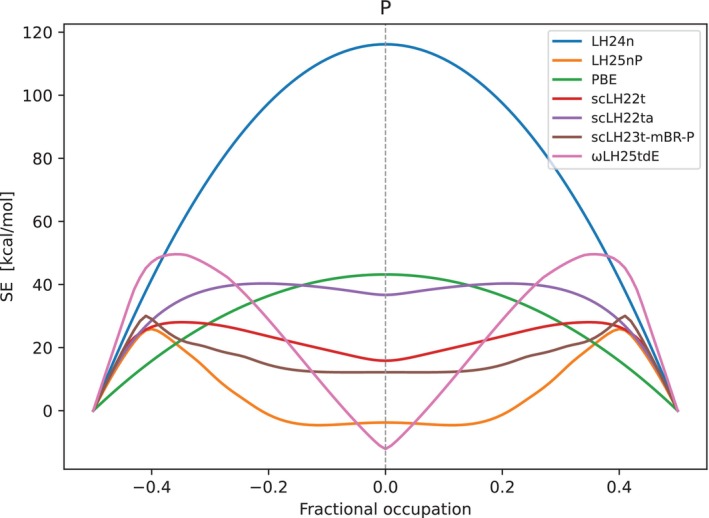
Energy deviations for the phosphorus atom as a function of fractional spin occupations ν in the 3p orbitals, interpolating between fully spin‐polarized and fully spin‐depolarized configurations. LH25nP‐D4 is compared with scLH23t‐mBR‐P, scLH22t, scLH22ta, ωLH25tdE, LH24n, and PBE.

The deviations of these spin‐restricted curves at dissociation are measures of FSE. We may alternatively look directly at the FSE values of the atoms used in LMF training. The difference between the energy computed with fully spin‐polarized (ν=±0.5) and fully spin‐depolarized atoms (ν=0.0) is the full FSE. Summed up for both atoms, it corresponds to the static‐correlation error at the spin‐restricted dissociation limit. But one can also look at intermediate situations with partially spin‐polarized atoms with values between the limits [14]. This provides us with an alternative view of situations with intermediately strong static correlation by plotting the energy deviations along a series of ν‐values, keeping in mind that the ideal performance should provide zero along the entire range [14]. Such plots for the phosphorus atom are shown in Figure 6, plots for other atoms are available in Figures S7, S8 in the Supporting Information. Consistent with the ${\text{P}}_{2}$ dissociation curve in Figure 3 above, LH25nP is closest to zero in the region of ν=0.0. The region of the unphysical maximum along the ${\text{P}}_{2}$ dissociation curve is likely connected to the regions around ν=±0.4 in the FSE plot, where the scLHs and scRSLHs all show maxima (as do B13, KP16/B13, DM21 and dRPA). Indeed, LH25nP has the smallest values at these peaks. ωLH25tdE has larger maxima and dives to slightly negative values near ν=0.0. We can associate the smaller HUMP10 values of scLH23t‐mBR‐P and scLH22ta to their somewhat less peaked curves which, however, show overall larger deviations from zero that lead to larger DISS10 MAEs. The “uncorrected” functionals LH24n and PBE exhibit the usual Gaussian‐shaped FSE curves with maxima at ν=0.0, reflecting their much larger FSEs and errors at the dissociation limit. Yet the curve for PBE suggests it to be well‐suited at intermediate ν values, likely due to its semi‐local hole that helps simulate some of the left‐right correlation, at the cost of large self‐interaction errors. Extending the analysis to other atoms (Figures S7 and S8) shows multiple maxima with LH25nP for some of the atoms, for example, N, O, and F. On the other hand the overall relatively flat curves for smaller absolute values of ν in most cases appear to be favorable for more strongly correlated situations.

A real‐life example where both delocalization and static‐correlation errors play a role is in unphysical spin‐symmetry breaking of certain open‐shell transition‐metal complexes, in particular from the 3d series [97, 98]. We have studied the hyperfine couplings of 3d transition‐metal complexes for decades, and had found that global hybrids with large EXX admixtures may help to provide improved core‐shell spin polarization but may also give rise to substantial spin contamination [99, 100, 101]. scRSLHs and scLHs have been shown to provide escape from this dilemma to variable extents as well [98]. It is of interest how LH25nP performs in this context. We have therefore applied it to the typical example complex ${\text{MnO}}_{3}$, which exhibits significant spin contamination and distorted dipolar hyperfine couplings with global, range‐separated or local hybrid functionals in the absence of correction terms. LH25nP‐D4 gives an $\left\langle S^{2}\right\rangle$ expectation value of 0.78 for this doublet‐state complex and a dipolar hyperfine coupling of 101 MHz (compared to an experimental value of 81 MHz), which is in the range of other scLHs (and scRSLHs). The isotropic hyperfine coupling is unrealistic, however (910 MHz). This is unsurprising given that the (unoptimized) core part of LH25nP exhibits too large EXX admixture over a too large spatial region (see, e.g., Figure 2). Yet the good spin‐densities obtained in the valence space for this and related complexes additionally confirm that LH25nP also should be expected to provide overall good density distributions when keeping in mind that the core region is so far unoptimized and not well defined. We note in passing that a) the optimization of a core LMF can be done more or less independently from the valence part, and b) this optimization requires self‐consistent calculations or core‐orbital energies or even TDDFT calculations of excitation energies [28]. Addition of a core LMF is beyond the scope of this work, however, and will be pursued elsewhere.

### 4.4 Evaluation of LH25nP‐D4 for Organometallic Transition‐Metal Reaction Energies and Barriers

To further assess the transferability of the LH25nP‐D4 model to systems far outside of its training domain, we evaluated its performance on benchmark sets involving organometallic transition‐metal complexes. These include the MOBH28 barrier set [69], the closed‐shell MOR41 reaction‐energy set [67], and the open‐shell ROST61 reaction‐energy set [68]. All data are available in the Supporting Information, Tables S5 to S8.

For the MOBH28 dataset, LH25nP‐D4 achieves a mean absolute error (MAE) of 2.25 kcal/mol, which is approximately 0.6 kcal/mol above ωLH25tdE‐D4 and 0.4 kcal/mol above LH24n‐D4. Such differences are likely within the accuracy range of the local coupled‐cluster reference data, and LH25nP‐D4 performs similarly as other types of hybrid functionals [69]. For MOR41, the MAE increases to 4.86 kcal/mol, clearly above ωLH25tdE‐D4 (2.47 kcal/mol) and LH24n‐D4 (approximately 3.65 kcal/mol). For the ROST61 dataset, LH25nP‐D4 gives an MAE of 5.44 kcal/mol, which is inferior to the best performing LH20t‐D4 (2.23 kcal/mol) and some other LHs and RSLHs albeit similar to other rung 3 or 4 functionals [68, 69]. This clearly shows deficiencies in the transferability of LH25nP‐D4 to transition‐metal reaction energies. LHs and RSLHs based on t‐LMFs perform clearly better here, even scLHs and scRSLHs. Obviously, an n‐LMF or nP‐LMF is much more flexible than a t‐LMF and should be able to perform well here too. This points to the need to extend the training of the nP‐LMF to such systems, while so far exclusively main‐group data have been used. We note in passing that the MOBH28, MOR41 and ROST61 data sets do not cover systems with large static correlation, as they have been specifically curated to exclude such reactions so as to present reliable single‐reference coupled‐cluster benchmark data. Interestingly, we found that removing iron and nickel complexes from the MOR41 and MOBH28 test sets (four reactions removed from each) significantly reduces the MAE to 2.2 and 1.9 kcal/mol, respectively. We note in passing that LH25nP‐D4 does not exhibit any problems with SCF convergence for such transition‐metal complexes. This contrasts with evaluations for DM21, where possible improvements for transition‐metal complexes with large static correlation cannot be harvested due to severe SCF convergence issues [22].

### 4.5 Evaluation of Molecular Structures

Despite the growing interest in machine‐learned density functionals, there is still limited data available regarding their performance beyond energy predictions. Molecular structures have been assessed in more detail only recently for the Skala deep‐neural‐network functional [55]. Other benchmark efforts for DM21 were limited only to small test sets and suffered from the lack of analytical gradients and overall high computational cost [102]. Analytical gradients for our LHs and RSLHs are straightforward to implement, and while somewhat larger prefactors make such gradient calculations slightly more costly within semi‐numerical integration implementations of the exact‐exchange energy density than for GHs or RSHs [103], structure optimizations are easy to carry out. Only minor code modifications were required to extend the implementation to the newly developed functionals based on an n‐LMF or nP‐LMF, and these improvements will be included in the upcoming release of Turbomole.

To evaluate performance of LH24n‐D4 and LH25nP‐D4 for molecular structures, we selected two very different gas‐phase structure benchmark test sets, LMGB35 [74] with small molecules composed only of light main‐group elements, and TMC32 [75] with more challenging metal‐ligand bond lengths in 3d transition‐metal complexes. For LMGB35, large def2‐QZVPPD basis sets were used (previous evaluations of Skala and DM21 used smaller def2‐TZVP basis sets). For TMC32, we used x2c‐TZVPall basis sets together with the scalar relativistic X2C method.

Individual results and statistical evaluations are given in Tables S9 to S11 in the Supporting Information. Starting with LMGB35, all functionals evaluated in Table S9 agree within less than one pm with the reference data. The MAE of LH24n‐D4 (0.45 pm) is comparable to that of LH20t‐D4 (0.42 pm), while that of LH25nP‐D4 is higher (0.72 pm). Using smaller def2‐TZVP basis sets (Table S10), as used in the evaluation of Skala [55], changes little, indicating that the structures of such small light main‐group compound are relatively uncritical.

The TMC32 3d transition‐metal‐ligand bond lengths are more challenging. A direct comparison with the earlier evaluation of DFAs in Reference [75] at non‐relativistic level has to be done with caution, as our data include scalar relativity. Interestingly, most hybrid functionals in Table S11 have a *negative* MSE of about −2 pm, the PBE GGA only by about −1 ppm. The latter functional also gives the smallest MAE (1.98 pm). LH24n‐D4 has a very small MSE of only −0.05 pm and the lowest MAE of 2.41 pm of the hybrid functionals. In contrast, LH25nP‐D4 is the only approach that *overestimates* the distances (MSE 1.59 pm) and has a relatively large MAE of 4.19 pm. As for the less convincing performance for organometallic transition‐metal energetics discussed above, this may reflect a lack of transferability of the nP‐LMF and points to the need of broader training, including also transition‐metal systems. Work along these lines is in progress.

Mixed‐valence (MV) systems require a particularly good balance between small delocalization and static‐correlation errors. Our MVO10 set (gas‐phase oxo systems; coupled cluster references and, for ${\text{V}}_{4}{\text{O}}_{10}^{.-}$, gas‐phase vibrational data) spans two extremes: ${\text{V}}_{4}{\text{O}}_{10}^{.-}$ with the extra electron delocalized over four V centers (the experimental structure has ${\text{D}}_{2d}$ symmetry; functionals with excessive EXX spuriously localize to ${\text{C}}_{s}$) and ${\text{Al}}_{2}{\text{O}}_{4}^{.-}$ with a hole localized on one terminal O in ${\text{C}}_{2v}$ (too small EXX admixture falsely yields a ${\text{D}}_{2h}$ structure with the hole delocalized over the two terminal O atoms). At CCSD(T) level there is also a very high‐lying ${\text{D}}_{2h}$ minimum with the hole on the bridging O atoms. No DFA gets both limits right; the closest are LH20t, LH23pt, and MN15, with the correct potential‐energy surface for ${\text{Al}}_{2}{\text{O}}_{4}^{.-}$, but still with ${\text{C}}_{s}$ → ${\text{D}}_{2d}$ barriers of 1.2, 1.8, 1.6 kcal/mol, respectively, for ${\text{V}}_{4}{\text{O}}_{10}^{.-}$ [28, 79, 104, 105].

We therefore tested the three NN‐based LHs LH24n‐B95, LH24n, and LH25nP for these two potential‐energy surfaces. For ${\text{Al}}_{2}{\text{O}}_{4}^{.-}$ all three functionals correctly recover the low‐lying localized ${\text{C}}_{2v}$ structures. Searches for the high‐lying bridge‐localized ${\text{D}}_{2h}$ minimum typically revert to a structure with the hole delocalized over the terminal O atoms (and therefore shorter Al‐Al distances). However, single‐point DFT energies at the CCSD(T) ${\text{D}}_{2h}$ minimum structure are reasonable, suggesting that the barrier has vanished or become too small to optimize a local minimum. Interestingly, LH25nP‐D4 locates this high‐lying bridge‐localized ${\text{D}}_{2h}$ minimum with the smaller grid (gridsize 3) used in LMF‐training but not with the larger grid (gridsize 7). For ${\text{V}}_{\text{4}}{\text{O}}_{10}^{.-}$, LH25nP gives a delocalized ${\text{D}}_{2d}$ structure with both grids, LH24n shows a tiny barrier (1.1 kcal/mol at gridsize 7, disappearing at gridsize 3), while LH24n‐B95 yields larger barriers (7.8 and 5.9 kcal/mol at gridsizes 7 and 3, respectively), indicating that replacing B95c with B97c correlation favors delocalization.

### 4.6 Ablation Studies

Designing neural networks inevitably involves a number of somewhat arbitrary choices, and an exhaustive grid search over architectures would be computationally prohibitive while unlikely to alter the main conclusions. Limited studies of the dependence on input features and size of neural network had been done for the earlier LH24n model (see Supporting Information of Reference [30]). Here we focus on two factors that appear most consequential: (i) the network width (64 vs. 128 units per hidden layer at fixed depth), and (ii) the representation of input features, comparing spin‐resolved (αβ) to spin‐summed (tot) descriptors.

To isolate these effects, we retrained otherwise identical models (same optimizer and hyperparameters; 400 epochs), varying only one aspect at a time, and initialized weights and biases with three distinct random seeds for each setup. Performance was evaluated on the Slim20‐GMTKN55 validation set [106] and on FSE10. Although FSE10 is part of the training objective (and thus not a strict validation metric), it provides insights into the flexibility of the model in dealing with strong correlations.

**FIGURE 7:**
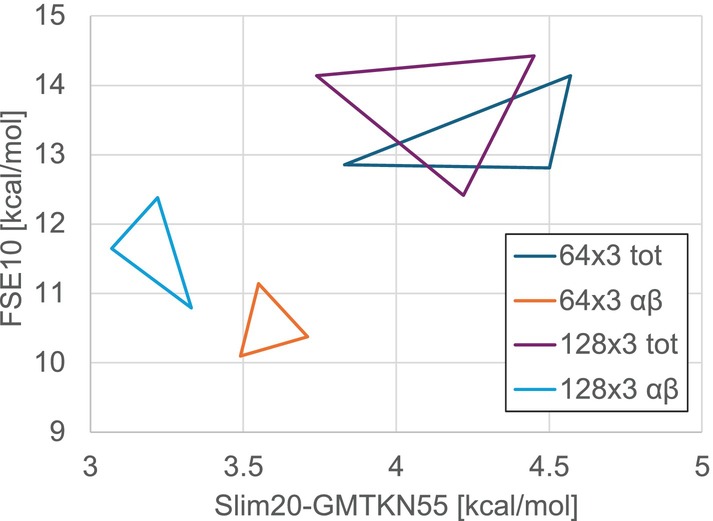
Deviations for Slim20‐GMTKN55 (X) versus FSE10 (Y) in kcal/mol for architectures 64 × 3 and 128 × 3 with two feature types: αβ = spin‐resolved features, tot = spin‐summed features. Each marker denotes one seed, points within the same variant are connected by a line.

The results (Figure 7) show consistent improvements when using spin‐resolved features: typical gains on Slim20 are on the order of 1 kcal/mol, with slight improvement (of up to 4 kcal/mol) for FSE10. Increasing the network width from 64 to 128 units yields smaller but still noticeable benefits for Slim20‐GMTKN55. We emphasize that these ablations are not exhaustive hyperparameter optimizations for each configuration. Nonetheless, they clearly capture the relevant trends.

### 4.7 Additional Technical Considerations

In view of the above grid dependence for the high‐lying ${\text{D}}_{2h}$ minimum of ${\text{Al}}_{2}{\text{O}}_{4}^{.-}$, we have evaluated grid dependencies further for LH25nP. As a specific grid has been used during the extensive neural‐network training, the question arises if grid‐overtraining may bias application of such functionals with different grid sizes. For the deep‐neural network functionals DM21 and Skala, this has been addressed by training on a mixture of grids to mitigate this dependence. We opted for a simpler strategy and trained our model using only the most commonly employed grids: grid size 3 for BH76, W4‐17, and FSE10, and grid size 4 for the diet‐GMTKN55 subset. The hope is that compared to deep‐neural‐network functionals the more limited machine learning of only the LMF with its well‐defined structure may reduce grid sensitivity.

To assess the extent of any residual grid dependence, we evaluated the performance of LH25nP‐D4 on BH76 and W4‐11 using grid sizes 3 to 5 (see Table S4 in the Supporting Information). As a reference, we also tested the human‐designed scLH23t‐mBR‐P‐D4 model, both with and without its CF, since the latter involves the Laplacian of the density, which is expected to be more grid‐sensitive. As anticipated, scLH23t‐mBR‐P‐D4 without the CF shows essentially no dependence on the integration grid. LH25nP‐D4 exhibits a larger sensitivity, particularly for W4‐11, but the variation remains moderate in comparison to scLH23t‐mBR‐P‐D4 with CF. These findings suggest that future iterations of neural‐network‐trained functionals could benefit from even limited mixed‐grid training to further reduce grid‐related artifacts.

A second technical consideration involves the use (or absence) of a CF to deal with the gauge problem. In contrast to scLH23t‐mBR‐P, LH25nP does not employ a CF. Instead, we rely entirely on the nP‐LMF to account for necessary corrections, as already found with the n‐LMF in LH24n‐B95 and LH24n. As a test we analyzed dissociation curves for two prototypical rare‐gas dimers, ${\text{Ne}}_{2}$ and ${\text{Ar}}_{2}$. As static correlation should be absent in such cases, reduction of the too repulsive energy curves in these two systems by a CF have previously been used to optimize the adjustable CF parameters [6, 32, 107]. The results (Figure S9 in the Supporting Information) show that LH25nP reproduces the nondynamical‐correlation‐free HF+B97c reference ${\text{Ar}}_{2}$ interaction curve with high accuracy without a CF. For ${\text{Ne}}_{2}$ the LH25nP curve goes very slightly below the reference curve. Clearly, the nP‐LMF suppresses gauge artefacts in such curves very well, likely due to its relatively high EXX admixture in the bonding region [29, 30], which also applies to the regions around the bond‐critical points in such non‐covalent interactions.

## 5 Conclusions

In the wide space between less empirical constructions of density functional approximations from mostly just exact physical constraints and completely black‐box empirical machine‐learned functionals, LH25nP as well as its predecessors LH24n and LH24n‐B95, occupy an intermediate space: only the position‐dependence (local mixing function) of exact‐exchange admixture in these local hybrid functionals has been trained as a still relatively shallow neural network (B97c parameters have been optimized separately). This allows insight into the local mixing function that is a 3D object we can visualize and analyze for any molecule. LH25nP goes beyond the other two functionals by incorporating into the local mixing function a strong‐correlation factor designed previously and training the neural‐network mixing function in its presence. In this way, LH25nP can escape the usual zero‐sum game between delocalization and strong‐correlation errors. It allows the proper spin‐restricted dissociation of covalent bonds due to the same adaptable property of its local mixing function as in other strong‐correlation corrected local hybrids: based on indicators for static correlation in coordinate space exact‐exchange admixture is reduced, even to the extent where it can become locally negative. LH25nP is the first local hybrid that achieves this with a neural‐network local mixing function, and it benefits from the latter by exhibiting low strong‐correlation errors and simultaneously the lowest deviations for the large GMTKN55 test suite, or of the even larger W4‐11RE reaction‐energy set, of a rung 4 functional so far. Tests of overtraining show that LH25nP‐D4 exhibits very little.

But evaluations for organometallic transition‐metal energetics and of transition‐metal‐ligand bond lengths suggest less favorable transferability to the realm of transition‐metal complexes compared to some of our local hybrids with simpler local mixing functions. This points clearly to the need for training on wider data sets including transition metals.

We have also demonstrated that analytical molecular gradients are straightforward to implement and apply for such LHs with neural‐network local mixing functions, in contrast to some other machine‐learned functionals like DM21. In general, the computational cost and scaling with basis‐set and system size for LH25nP‐D4 is the same as for other LHs in the same setting with semi‐numerical integration of EXX energy densities. This makes it straightforward to apply such functionals to a wide range of questions. Ongoing work in our lab concentrates on machine‐learned LMFs that directly incorporate strong‐correlation effects without a human‐designed strong‐correlation factor, and on transferring these ideas to range‐separated local hybrids.

## Supporting information




**Data S1.** Supporting Information.

## Data Availability

The data that supports the findings of this study are in part available in the Supporting Information of this article. The weights and biases of the trained LMF are available at https://doi.org/10.5281/zenodo.16988891. The neural network training code nLMFs and the B97opt code for training the linear parameters of B97 correlation are publicly available at https://github.com/awodynski/nLMFs_batches and https://github.com/awodynski/B97opt, respectively.
