# Supplementary material for: Local‐Hybrid Functional With a Composite Local Mixing Function Built From a Neural Network and a Strong‐Correlation Model
Source: J Comput Chem. 2026 Jan 24;47(3):e70294. doi: 10.1002/jcc.70294 (PMC12831636; doi:10.1002/jcc.70294)
Supplement: Supplementary file 1 — Data S1. Supporting Information. [file JCC-47-0-s001.pdf]

Supplementary Information: Local-Hybrid Functional with a Composite Local Mixing Function Built from a Neural Network and a Strong-Correlation Model

Artur Wodyński,* Martin Kaupp*

Correspondence.
Artur Wodyński, Technische Universität Berlin, Institute of Chemistry, Theoretical Chemistry/Quantum Chemistry, Sekr. C7, Straße des 17. Juni 135, 10623 Berlin, Germany. Email: artur.wodynski@tu-berlin.de.
Martin Kaupp, Technische Universität Berlin, Institute of Chemistry, Theoretical Chemistry/Quantum Chemistry, Sekr. C7, Straße des 17. Juni 135, 10623 Berlin, Germany. Email: martin.kaupp@tu-berlin.de.

S1 Details of implementation

S1.1 Neural-network local mixing function in LH25nP

We employ a compact MLP to produce a spin-symmetrized LMF, attenuated by the pre-defined strong-correlation factor $q_{\mathrm{AC}}(\mathbf{r})$.

Inputs and scaling. The feature vector is

$$\mathbf{x}(\mathbf{r}) = \big(n_\alpha, n_\beta,\ |\nabla n_\alpha|^2,\ \nabla n_\alpha \cdot \nabla n_\beta,\ |\nabla n_\beta|^2,\ e_\alpha,\ e_\beta,\ \tau_\alpha,\ \tau_\beta\big) \in \mathbb{R}^9.$$

Each component is transformed element-wise to stabilize magnitudes:

$$\mathbf{x}_s(\mathbf{r}) = \mathrm{sgn}\big(\mathbf{x}(\mathbf{r})\big)\ \log\big(1 + |\mathbf{x}(\mathbf{r})|\big).$$

Network. A three-hidden-layer MLP maps $\mathbf{x}_s(\mathbf{r})$ to a scalar $\tilde{a}(\mathbf{r}) \in (-1, 1)$:

$$\begin{aligned}
\mathbf{h}_1(\mathbf{r}) &= \mathrm{GELU}(\mathbf{W}_1\, \mathbf{x}_s(\mathbf{r}) + \mathbf{b}_1)\,, && \mathbf{W}_1 \in \mathbb{R}^{128\times 9},\ \mathbf{b}_1 \in \mathbb{R}^{128},\\
\mathbf{h}_2(\mathbf{r}) &= \mathrm{GELU}(\mathbf{W}_2\, \mathbf{h}_1(\mathbf{r}) + \mathbf{b}_2)\,, && \mathbf{W}_2 \in \mathbb{R}^{128\times 128},\ \mathbf{b}_2 \in \mathbb{R}^{128},\\
\mathbf{h}_3(\mathbf{r}) &= \mathrm{GELU}(\mathbf{W}_3\, \mathbf{h}_2(\mathbf{r}) + \mathbf{b}_3)\,, && \mathbf{W}_3 \in \mathbb{R}^{128\times 128},\ \mathbf{b}_3 \in \mathbb{R}^{128},\\
\tilde{a}(\mathbf{r}) &= \sigma(\mathbf{W}_4\, \mathbf{h}_3(\mathbf{r}) + \mathbf{b}_4)\,, && \mathbf{W}_4 \in \mathbb{R}^{1\times 128},\ \mathbf{b}_4 \in \mathbb{R}^{1},
\end{aligned}$$

with GELU activations[S1]:

$$\mathrm{GELU}(x) = x \cdot \frac{1}{2}\left[1 + \mathrm{erf}\left(\frac{x}{\sqrt{2}}\right)\right], \tag{S1}$$

and a shifted sigmoid[S2] output:

$$\sigma(x) = \frac{2}{1 + e^{-x}} - 1. \tag{S2}$$

Number of total trainable parameters: 34433.

Spin symmetrization. To enforce invariance under $\alpha \leftrightarrow \beta$ exchange, the network is evaluated twice, with (α, β) and with swapped (β, α) inputs, and the results are averaged[S3]:

$$a_{av}(\mathbf{r}) = \tfrac{1}{2}\Big[\tilde{a}\big(\mathbf{x}_s^{(\alpha,\beta)}(\mathbf{r})\big) + \tilde{a}\big(\mathbf{x}_s^{(\beta,\alpha)}(\mathbf{r})\big)\Big].$$

Strong-correlation attenuation. The LH25nP local mixing function used in the exchange energy density is the symmetrized MLP output multiplied by the analytic attenuation function $q_{\mathrm{AC}}(\mathbf{r})$:

$$\text{nP-LMF}(\mathbf{r}) = 1 - 2q_{\mathrm{AC}}(\mathbf{r})\,(1 - a_{av}(\mathbf{r})),$$

The Padé-form strong-correlation factor $q_{\mathrm{AC}}(\mathbf{r})$ is defined as

$$q_{AC}(z(\mathbf{r})) = 0.5 + d \cdot \frac{k z^i(\mathbf{r})}{1 + k z^i(\mathbf{r})}, \tag{S3}$$

where

$$z(\mathbf{r}) = \max\left(\frac{\sum_\sigma e_{X,\sigma}^{\mathrm{mBR}}(\mathbf{r})}{\sum_\sigma e_{X,\sigma}^{\mathrm{ex}}(\mathbf{r})} - 1, 0\right). \tag{S4}$$

d, k, and i are adjustable parameters. For practical reasons, we use parameters p_1 and p_2 in place of k and i. These control the damping and saturation of q_{AC}, with $i = -4 \cdot \ln(3)/\ln(p_1/p_2)$ and $k = -9 \cdot p_2^{-i}$.

The mBR[S4] exchange model is given by

$$e_{X,\sigma}^{\mathrm{mBR}} = -\pi^{1/3}\rho^{4/3}\left(\frac{\mathrm{e}^{y_\sigma/3}}{y_\sigma}\right)\left[1 - \mathrm{e}^{-y_\sigma} - \frac{1}{2}y_\sigma \mathrm{e}^{-y_\sigma}\right], \tag{S5}$$

where y_σ is calculated by solving

$$\frac{y_\sigma \mathrm{e}^{-2y_\sigma/3}}{(y_\sigma - 2)} = \frac{2}{3}\pi^{2/3}\frac{\rho_\sigma^{5/3}}{Q_\sigma^{mBR}} \tag{S6}$$

with

$$\begin{aligned} Q_\sigma^{mBR}(\boldsymbol{r}) = \quad & \frac{1}{6}\left[2\left(\gamma^2 - \gamma + \frac{1}{2}\right)\nabla^2\rho_\sigma(\boldsymbol{r}) + \right. \\ & \left. \frac{6}{5}k_\sigma^2(\boldsymbol{r})\rho_\sigma(\boldsymbol{r})(f_\sigma^2(\boldsymbol{r}) - 1) - 2D_\sigma^m(\boldsymbol{r})\right] \end{aligned} \tag{S7}$$

$$\begin{aligned} f_\sigma(\boldsymbol{r}) = \quad & \left[1 + 10\frac{70}{27}\frac{1}{4(6\pi^2)^{\frac{2}{3}}}(2\gamma - 1)^2\frac{|\nabla\rho_\sigma(\boldsymbol{r})|^2}{\rho_\sigma^{8/3}(\boldsymbol{r})}\right. \\ & \left. + \frac{\beta}{16(6\pi^2)^{\frac{4}{3}}}(2\gamma - 1)^4\frac{|\nabla\rho_\sigma(\boldsymbol{r})|^4}{\rho_\sigma^{16/3}(\boldsymbol{r})}\right]^{\frac{1}{10}} \end{aligned} \tag{S8}$$

$$D_\sigma^m(\boldsymbol{r}) = 2\tau_\sigma(\boldsymbol{r}) - 2(2\gamma - 1)^2\tau_{W,\sigma}(\boldsymbol{r}) \tag{S9}$$

and

$$k_\sigma(\boldsymbol{r}) = \sqrt[3]{6\pi^2\rho_\sigma(\boldsymbol{r})}. \tag{S10}$$

$\gamma = 0.878795$ and $\beta = 17.388054$ are adjustable parameters. Note that the version used here employs the full density Laplacian instead of the approximated form used in the original mBR functional.

Differentiability and SCF. All operations (scaling, affine layers, GELU, sigmoid, averaging, and multiplication by q_{AC}) are differentiable; functional derivatives for the XC potential follow from the chain rule within the same computational graph, aiding stable SCF convergence.

S1.2 Details of loading weights and biases

Algorithm 1 Loading weights and biases from file available at: https://doi.org/10.5281/zenodo.16988891

```
Initialize num_layers = 4 , num_neurons_layer = 128, and num_features = 7
Initialize an array for weights: weights[num_layers, num_neurons, num_neurons_layer]
Initialize an array for biases: biases[num_layers, num_neurons_layer]
Open file for reading
for each layer L from 1 to num_layers do
    if L is 1 (first layer) then
        Set num_inputs = num_features
    else
        Set num_inputs = num_neurons_layer
    end if
    if L is the num_layers (last layer) then
        Set num_neurons = 1
    else
        Set num_neurons = num_neurons_layer
    end if
    for each neuron N in layer L do
        for each input I to neuron N do
            Read weight from file and store in weights[L, N, I]
        end for
        Read bias for neuron N and store in biases[L, N]
    end for
end for
Close file
```

S1.3 Model exchange energy density

The PBE exchange functional[S5] is given by

$$e_X^{\text{PBE}} = -C_{\text{X}}\rho^{4/3}(\mathbf{r})(1+\kappa-\kappa/\left(1+\mu s^2/\kappa\right)), \tag{S11}$$

where $C_{\text{X}} = \frac{3}{4}\left(\frac{6}{\pi}\right)^{1/3}$, $s^2 = |\nabla\rho(\boldsymbol{r})|^2/(4\cdot 3^{2/3}\pi^{4/3}\rho^{8/3})$, $\mu = 0.21951$ and $\kappa = 0.804$. Note that the total exchange energy for a spin-polarized system is obtained by the spin-scaling relationship

$$e_X^{\text{PBE}}(\rho_\sigma, \rho_{-\sigma}) = (e_X^{\text{PBE}}(2\rho_\sigma) + e_X^{\text{PBE}}(2\rho_{-\sigma}))/2. \tag{S12}$$

S1.4 B97c dynamical correlation

The spatially integrated dynamical correlation energy is given by

$$e_{\text{B97c}}(\mathbf{r}) = e_{\text{B97c}}^{opp}(\mathbf{r}) + \sum_\sigma e_{\text{B97c}}^{\sigma\sigma}(\mathbf{r}), \tag{S13}$$

where the opposite-spin contribution is defined as

$$e_{\text{B97c}}^{opp}(\mathbf{r}) = \sum_{i=0,m} d_{opp,i}\left(\frac{c_{opp}(\chi_\alpha^2(\mathbf{r})+\chi_\beta^2(\mathbf{r}))}{1+c_{opp}(\chi_\alpha^2(\mathbf{r})+\chi_\beta^2(\mathbf{r}))}\right)^i \cdot e_{c,opp}^{\text{UEG}}(\mathbf{r}), \tag{S14}$$

and the same-spin contribution is defined as

$$e^{\sigma\sigma}_{\mathrm{B97c}}(\mathbf{r}) = \sum_{i=0,m} d_{\sigma\sigma,i} \left(\frac{c_{\sigma\sigma}(\chi^2_\sigma(\mathbf{r}))}{1 + c_{\sigma\sigma}(\chi^2_\sigma(\mathbf{r}))} \right)^i \cdot \alpha_\sigma \cdot e^{\mathrm{UEG}}_{c,\sigma\sigma}(\mathbf{r}), \tag{S15}$$

where $\chi_\sigma(\mathbf{r}) = \frac{(\nabla^{\mathrm{T}}\rho_\sigma(\mathbf{r})\nabla\rho_\sigma(\mathbf{r}))^{1/2}}{\rho_\sigma^{4/3}(\mathbf{r})}$ is the reduced density gradient and $\alpha_\sigma(\mathbf{r}) = \frac{\tau_\sigma(\mathbf{r})-\tau_w(\mathbf{r})}{\tau_{\mathrm{UEG},\sigma}(\mathbf{r})}$ is used as a self-correlation correction for the same-spin contribution. $e^{\mathrm{UEG}}_{c,opp}$ and $e^{\mathrm{UEG}}_{c,\sigma\sigma}$ are PW92[S6] LDA opposite- and same-spin contributions, respectively.

S1.5 Parameters of LH25nP-D4

Table S1. Optimized parameters of LH25nP-D4 in addition to the n-LMF (see above), including the associated D4 dispersion corrections.[a]

	parameter	Value
$q^{\mathrm{Pade}}_{\mathrm{AC}}(\mathbf{r})$	d	0.725653
	p_1	0.089933
	p_2	0.134653
	γ	0.878795
	β	17.388054
B97c	c_{opp}	0.004987
	$c_{\sigma\sigma}$	0.09544
	$d_{\mathrm{opp},0}$	**1.41734321**
	$d_{\mathrm{opp},1}$	**-2.85105747**
	$d_{\mathrm{opp},2}$	**3.22104274**
	$d_{\mathrm{opp},3}$	**-1.79413764**
	$d_{\sigma\sigma,0}$	**0.15449654**
	$d_{\sigma\sigma,1}$	**0.01689284**
	$d_{\sigma\sigma,2}$	**-0.19745769**
	$d_{\sigma\sigma,3}$	**0.02150682**
D4	s_8	**0.42366769**
	a_1	**0.35**
	a_2	**4.70**

[a]Values of parameters optimized in this work are given in bold print. The others have been retained from the scLH23t-mBR-P functional.[S7]

S2 Additional Results

Table S2. Mean absolute deviations (MAD) in kcal/mol with selected local hybrid and range-separated local hybrid functionals (including D4 corrections) for subsets of the GMTKN55 test suite.

	ωLH25tdE-D4	LH24n-D4	LH25nP-D4
ACONF	0.03	0.08	0.03
ADIM6	0.11	0.12	0.05
AHB21	0.36	0.25	0.78
AL2X6	1.89	1.07	1.10
ALK8	3.73	1.53	4.84
ALKBDE10	3.12	3.91	3.84
AMINO20x4	0.20	0.14	0.14
BH76	1.00	1.20	0.79
BH76RC	1.25	0.96	0.82
BHDIV10	0.77	0.85	1.04
BHPERI	0.67	0.99	0.64
BHROT27	0.32	0.32	0.27
BSR36	0.34	1.99	0.44
BUT14DIOL	0.06	0.07	0.08
C60ISO	1.02	5.27	15.01
CARBHB12	0.26	0.50	0.24
CDIE20	0.38	0.55	0.25
CHB6	0.92	0.55	1.09
DARC	0.64	0.95	0.47
DC13	5.18	4.07	2.66
DIPCS10	7.55	6.13	11.70
FH51	1.21	1.66	0.89
G21EA	1.57	1.78	2.61
G21IP	3.26	3.25	5.42
G2RC	2.00	2.79	1.60
HAL59	0.27	0.35	0.27
HEAVY28	0.13	0.28	0.10
HEAVYSB11	1.01	0.97	1.88
ICONF	0.24	0.18	0.16
IDISP	0.87	1.12	0.86
IL16	1.41	1.20	1.24
INV24	1.07	1.06	0.95
ISO34	0.95	0.71	0.43
ISOL24	1.82	1.41	1.31
MB16-43	14.22	20.25	20.24
MCONF	0.13	0.11	0.13
NBPRC	1.11	1.57	1.11
PA26	1.09	1.91	3.67
PArel	0.48	0.73	0.50
PCONF21	0.21	0.20	0.36
PNICO23	0.18	0.30	0.17
PX13	1.71	1.21	3.97
RC21	1.52	1.62	1.56
RG18	0.08	0.06	0.04
RSE43	0.34	0.44	0.36
S22	0.20	0.17	0.19
S66	0.16	0.12	0.13
SCONF	0.14	0.15	0.14
SIE4x4	6.06	6.90	5.85
TAUT15	0.49	0.59	0.56
UPU23	0.74	0.83	0.41
W4-11	2.89	3.61	2.10
WATER27	1.27	0.91	1.35
WCPT18	1.08	1.60	1.26
YBDE18	1.24	1.89	1.50

Table S3. Deviations in kcal/mol for the atomization energy of C_2 with different functionals

	deviation in kcal/mol
PBE-D3(BJ)	-2.70
PBE0-D3(BJ)	-26.13
LH20t-D4	-32.64
scLH22ta-D4	-13.97
scLH22t-D4	-12.79
ωLH25tdE	-4.19
LH24n-D4	-37.81
LH25nP-D4	8.52
DSD-PBEB95-D3(BJ)	-10.75
DSD-PBEP86-D3(BJ)	-9.90
ωDH25-D4	-14.35

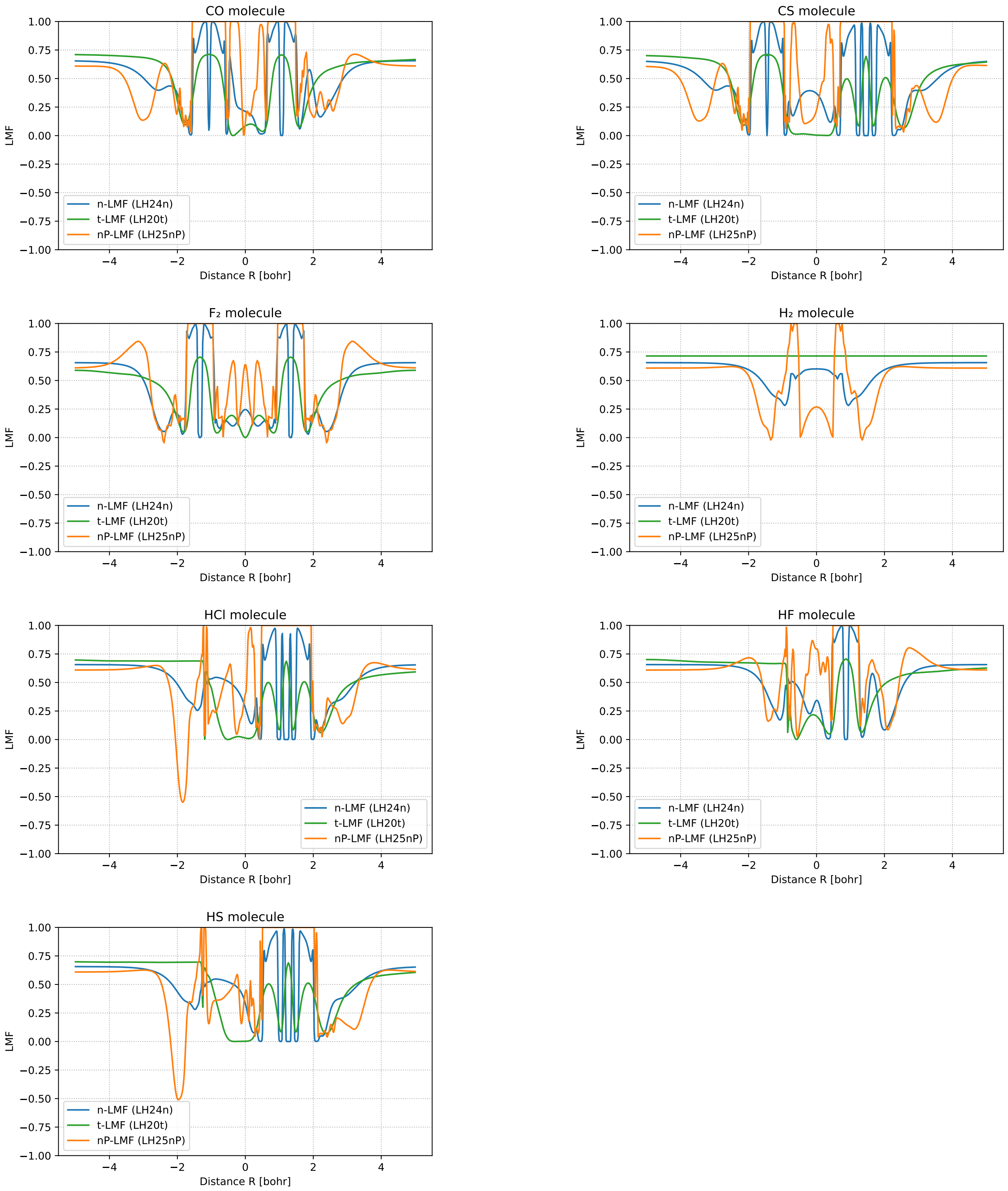

Figure S1. Comparison of plots of the nP-LMF of LH25nP, the n-LMF of LH24n, and the t-LMF of LH20t for selected diatomic molecules along the bond axis (def2-QZVP basis sets).

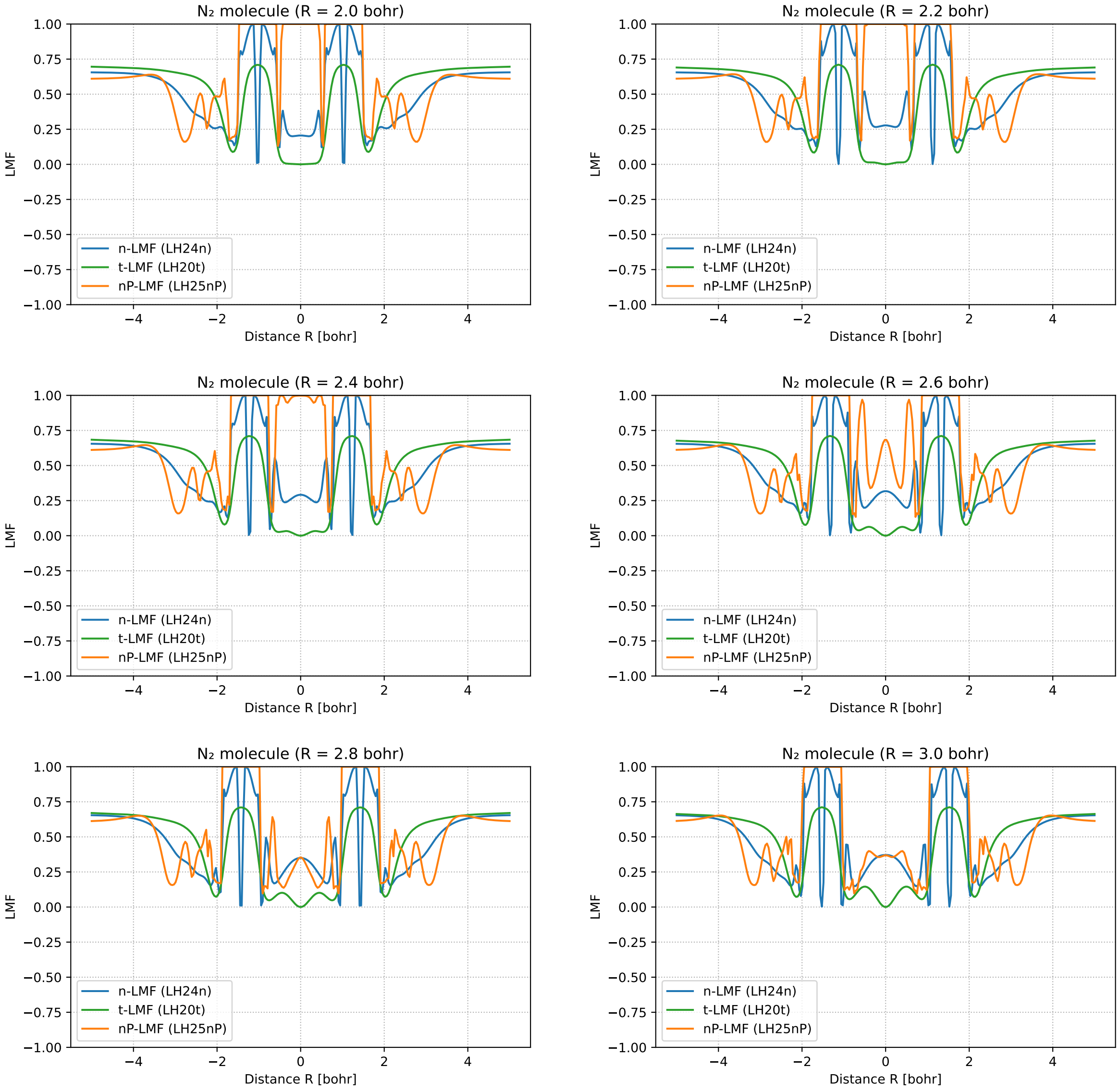

Figure S2. Comparison of plots of the nP-LMF of LH25nP, the n-LMF of LH24n, and the t-LMF of LH20t for N_2 at different (stretched) bond lengths (def2-QZVP basis sets). See Figure S6 below for still longer distances.

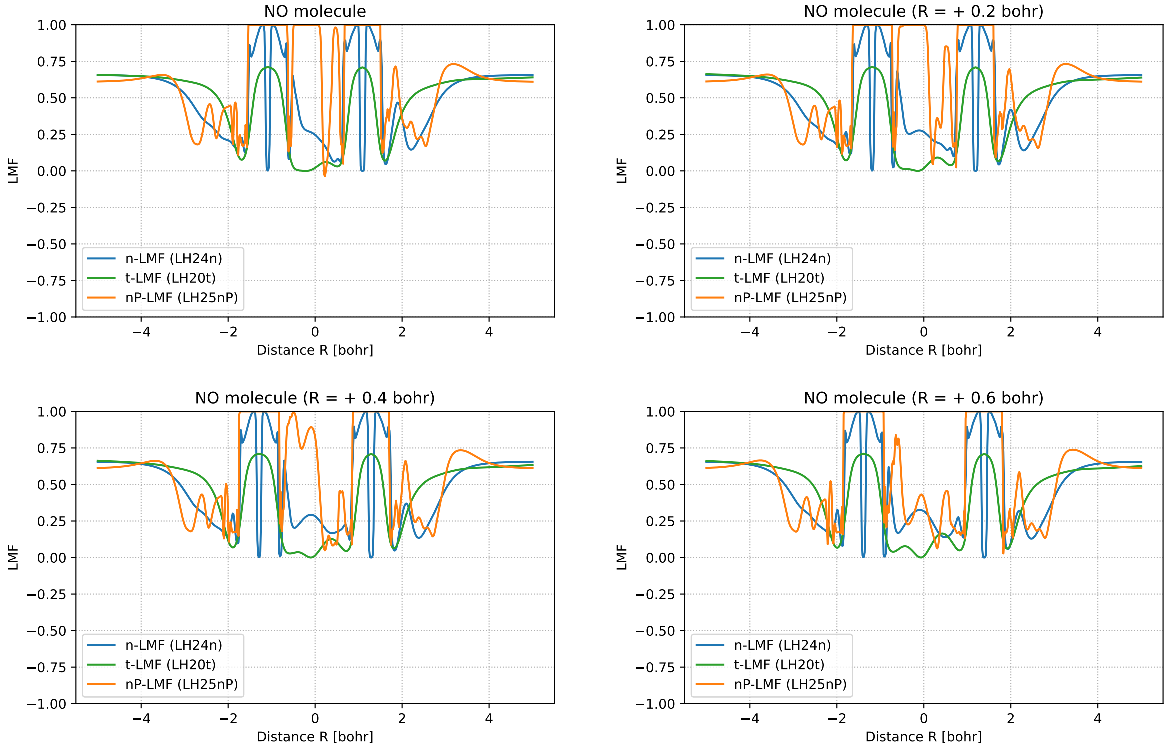

Figure S3. Comparison of plots of the nP-LMF of LH25nP, the n-LMF of LH24n, and the t-LMF of LH20t for NO at different (stretched) bond lengths (def2-QZVP basis sets).

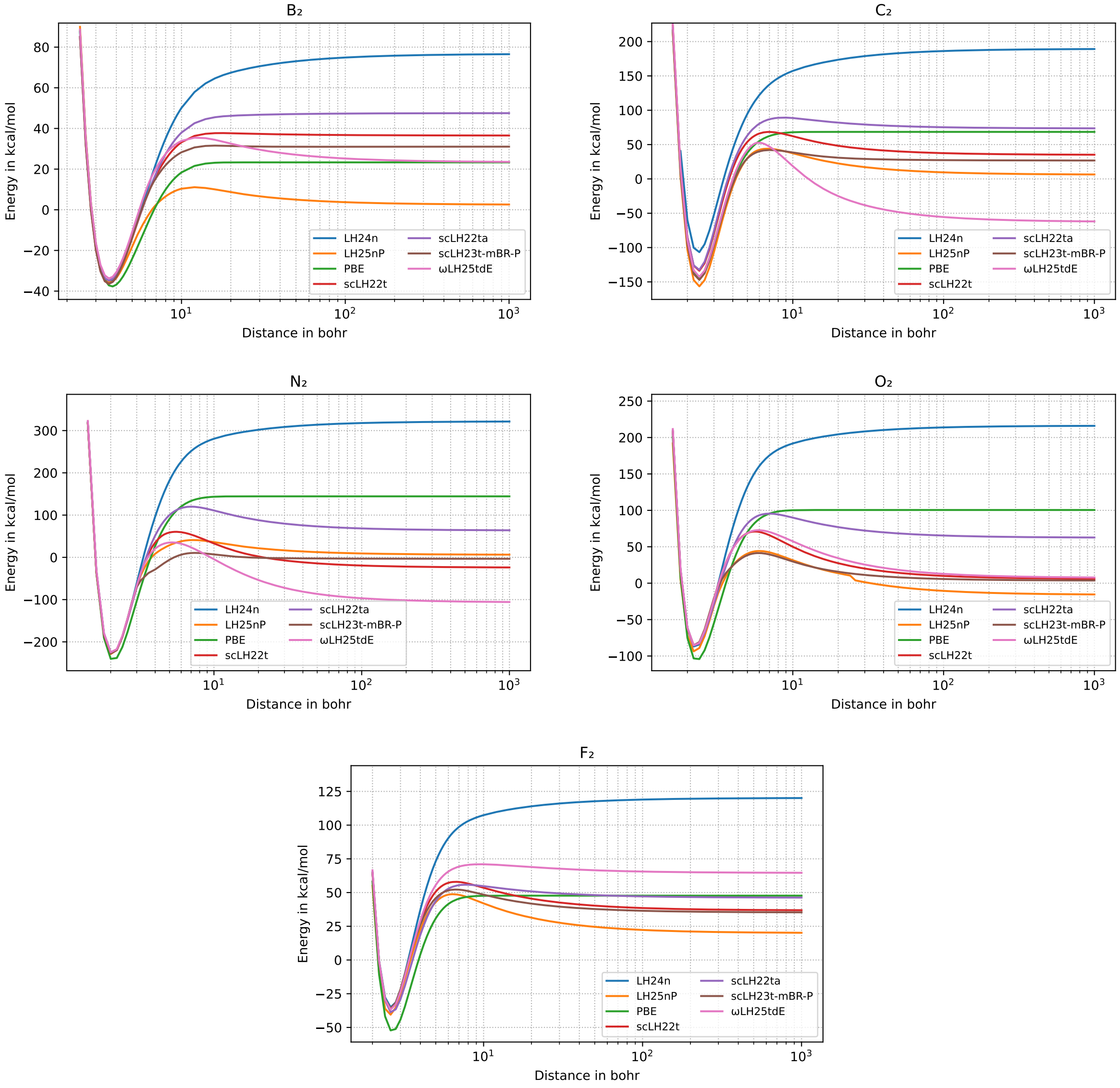

Figure S4. Dissociation curves for second-period diatomic molecules calculated in the lowest closed-shell singlet state at spin-restricted DFT level with different density functionals (def2-QZVPPD basis sets, gridsize 4 with additional diffuse grid).

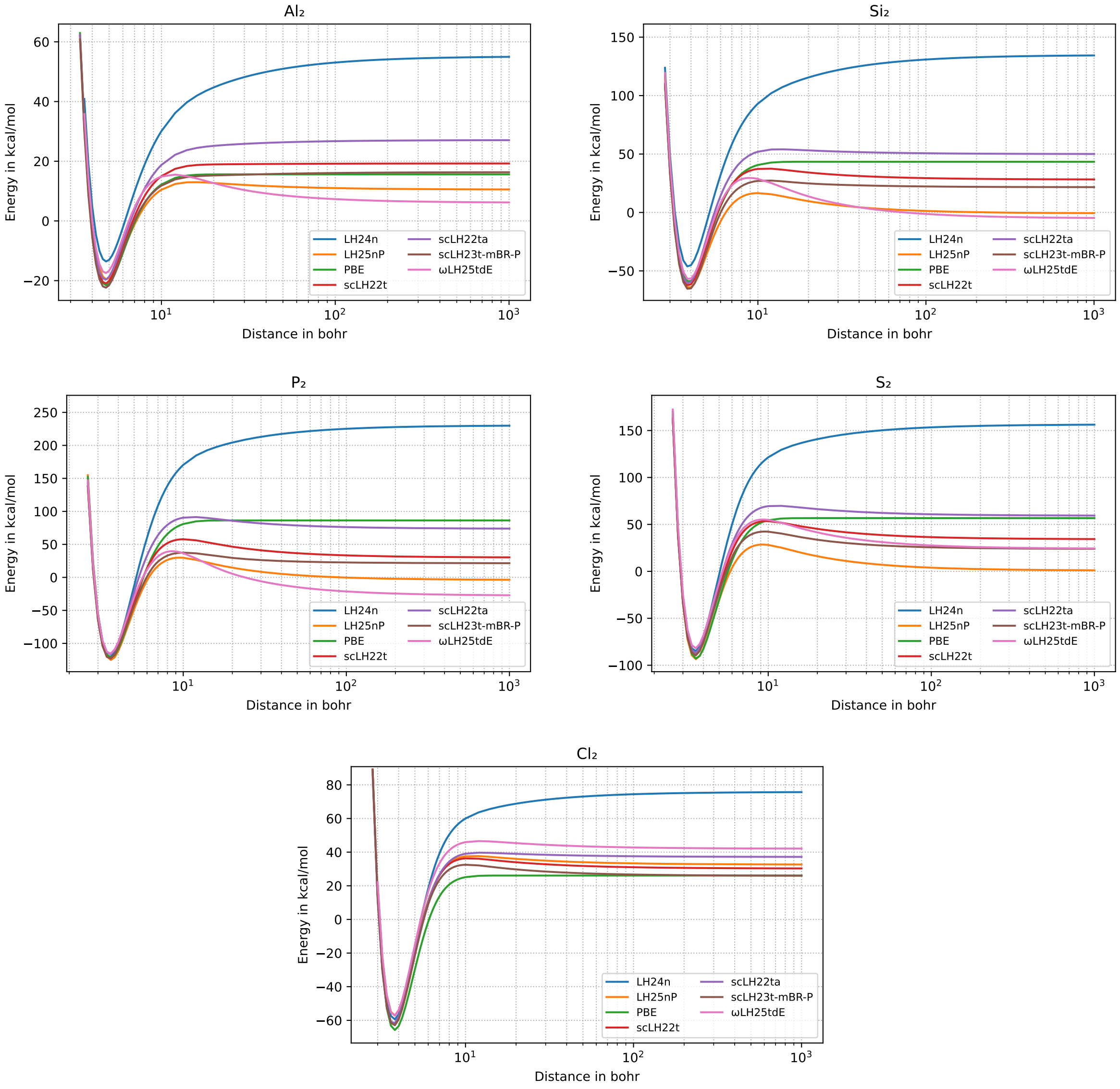

Figure S5. Dissociation curves for third-period diatomic molecules calculated in the lowest closed-shell singlet state at spin-restricted DFT level with different density functionals (def2-QZVPPD basis sets, gridsize 4 with additional diffuse grid).

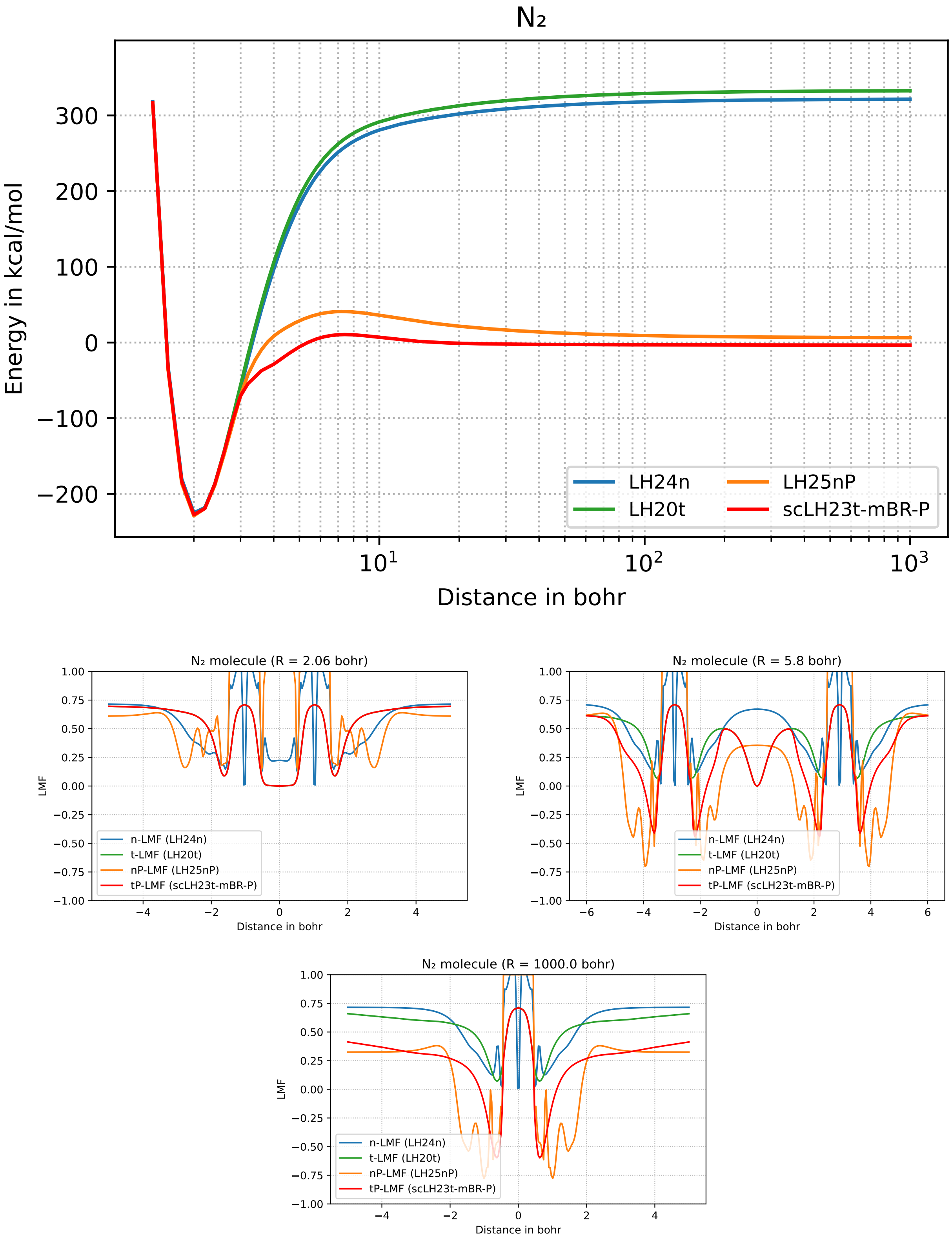

Figure S6. Dissociation curves for N_2 calculated at spin-restricted DFT level with selected LHs, and plots of the corresponding LMFs at equilibrium (2.06 bohr), at intermediate distance (5.8 bohr), and near asymptotic limit (1000.0 bohr; here only one nitrogen atom is inside the plot).

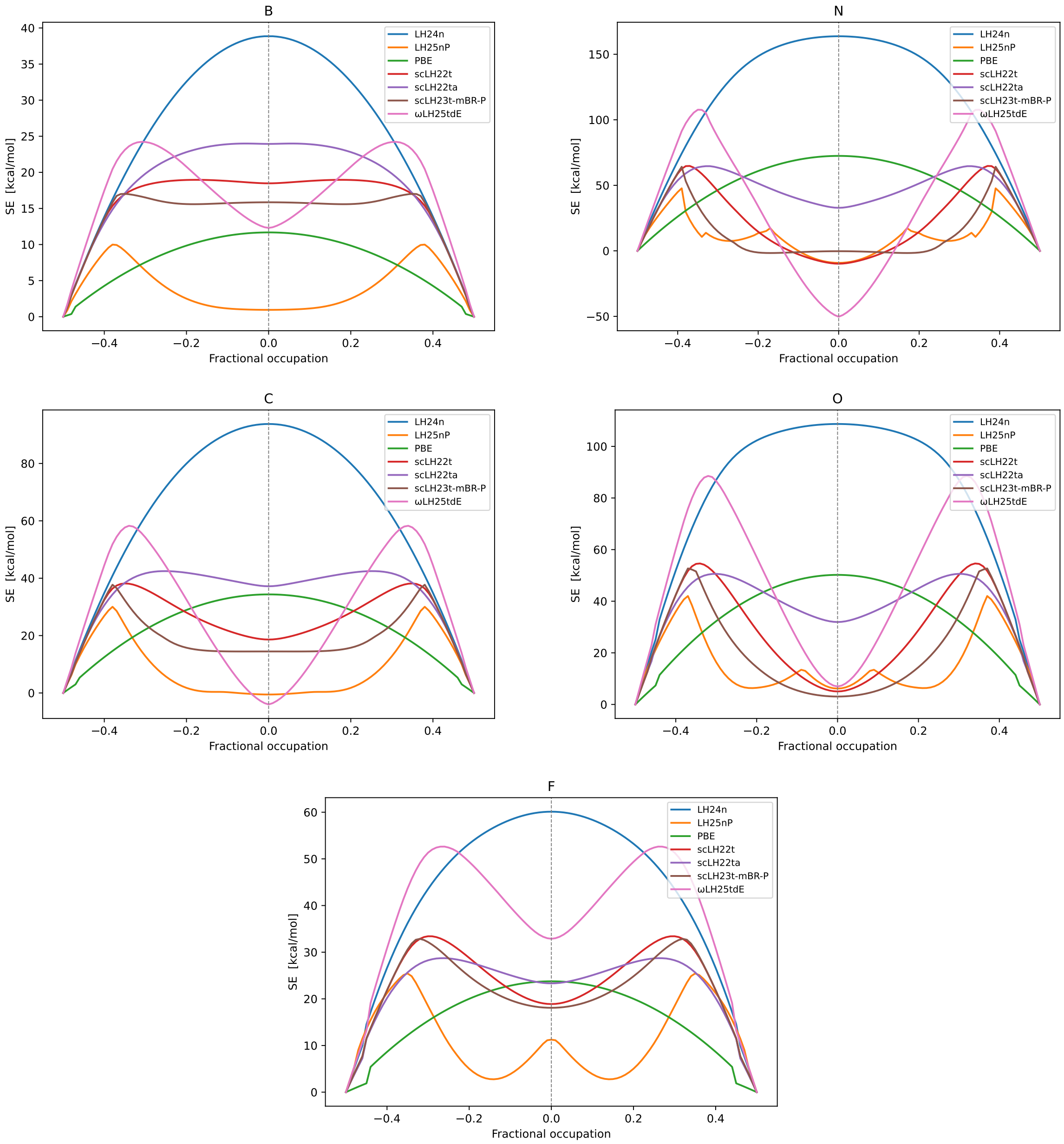

Figure S7. Fractional-spin-error curves of second-period atoms calculated with different functionals (def2-TZVP basis sets).

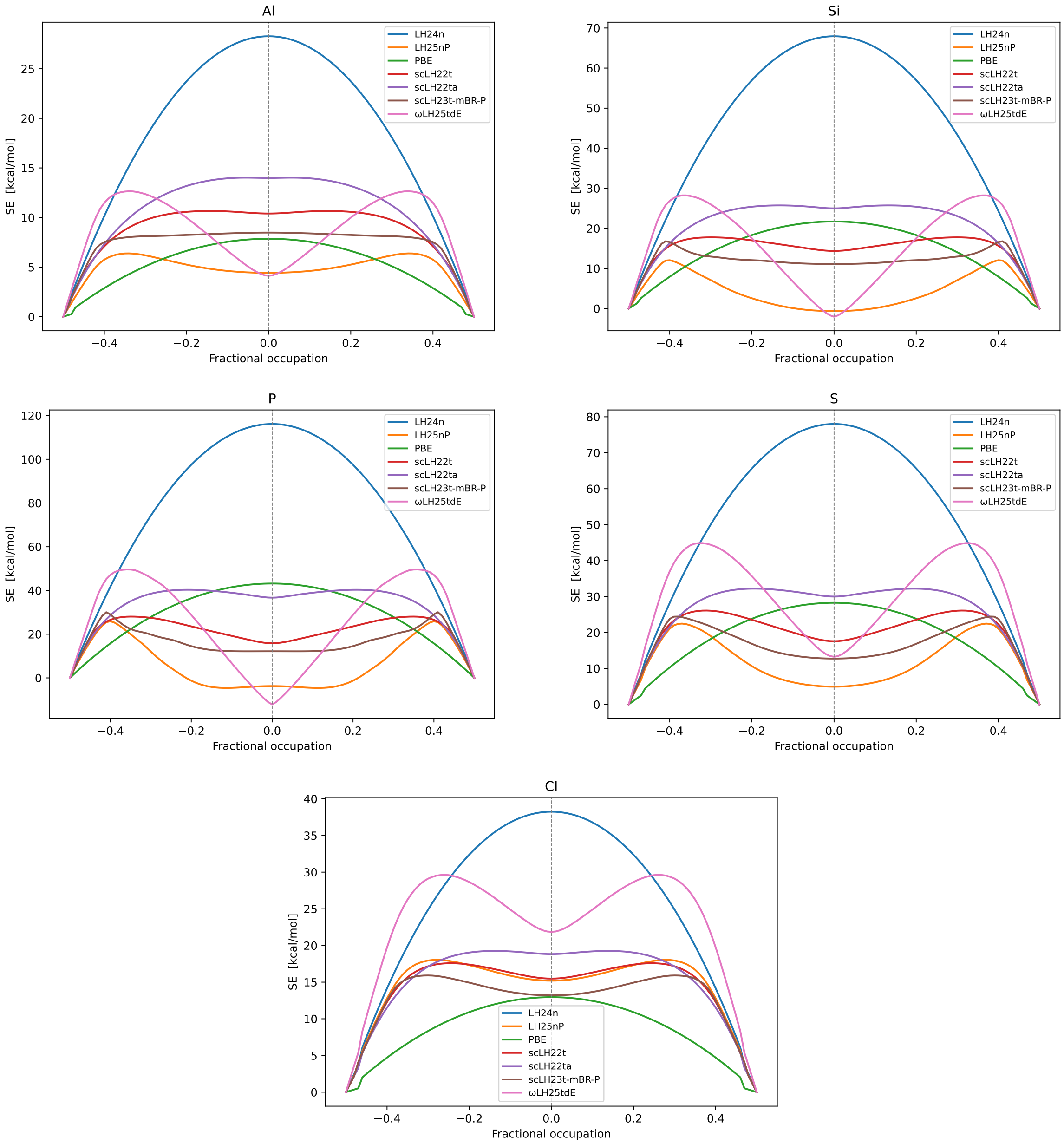

Figure S8. Fractional-spin-error curves of third-period atoms calculated with different functionals (def2-TZVP basis sets).

		BH76	W4-11
gridsize 3	scLH23t-mBR-P-D4 (no calib)	1.412	6.952
	scLH23t-mBR-P-D4	2.148	2.587
	LH25nP-D4	0.711	1.767
gridsize 4	scLH23t-mBR-P-D4 (no calib)	1.413	6.947
	scLH23t-mBR-P-D4	2.152	3.047
	LH25nP-D4	0.819	2.413
gridsize 5	scLH23t-mBR-P-D4 (no calib)	1.411	6.947
	scLH23t-mBR-P-D4	2.154	2.592
	LH25nP-D4	0.730	2.655

Table S4. Grid-size dependence of MAEs for the BH76 and W4-11 test sets using both human-designed and neural-network-trained functionals. For scLH23t-mBR-P-D4, "no calib" means the calibration function has been removed.

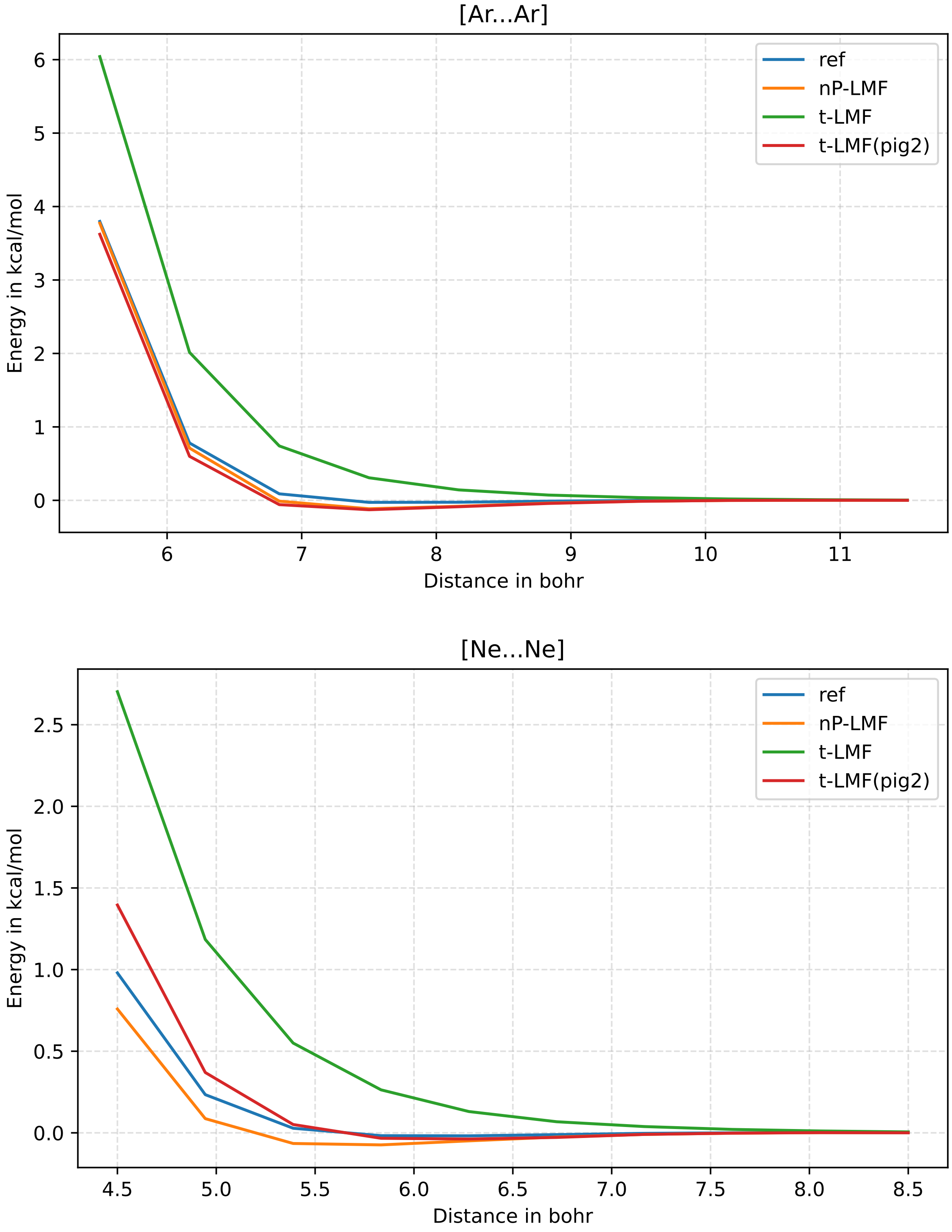

Figure S9. Dissociation curves for the Ar_2 and Ne_2 dimers, where nondynamical correlation is expected to be negligible. Deviations from the HF+B97c reference curve indicate gauge problems of the exchange-energy densities. Results for the t-LMF of LH20t are shown with and without the pig2 CF, no CF is used for the nP-LMF of LH25nP.

Table S5. Comparison of MOR41 reaction-energy results and statistical data in kcal/mol for selected functionals.

Reaction	Reference	LH20t-D4	LH24n-D4	ωLH25tdE-D4	LH25nP-D4
1	-43.10	-42.30	-38.11	-40.42	-37.01
2	-46.60	-44.12	-36.65	-43.57	-32.49
3	-27.60	-23.41	-14.23	-23.21	-8.44
4	-62.50	-64.08	-61.06	-62.54	-60.54
5	3.70	-1.96	1.31	4.05	4.73
6	-23.20	-17.58	-17.28	-18.64	-17.60
7	-16.20	-12.73	-14.12	-11.31	-14.21
8	-17.20	-8.21	-12.65	-12.97	-12.10
9	-18.70	-5.52	-12.25	-17.39	-16.67
10	-22.60	-16.63	-13.63	-20.73	-17.29
11	27.00	29.29	33.89	30.00	34.16
12	-29.80	-29.60	-25.45	-27.76	-23.53
13	-43.20	-43.49	-39.66	-41.78	-37.08
14	-52.00	-51.61	-42.24	-47.84	-40.34
15	-4.10	-4.79	-17.72	-6.70	-27.20
16	-39.80	-39.73	-38.50	-39.25	-37.86
17	-16.10	-14.27	-15.46	-15.24	-12.88
18	-34.20	-32.73	-31.45	-29.39	-30.50
19	-40.10	-39.27	-38.05	-36.09	-35.12
20	-30.20	-30.32	-30.00	-27.91	-25.99
21	-15.10	-16.93	-17.05	-16.32	-17.70
22	-35.90	-34.97	-30.42	-23.87	-27.33
23	-55.00	-52.26	-50.47	-46.14	-49.57
24	-41.60	-41.54	-40.08	-38.93	-38.96
25	-45.90	-45.82	-43.99	-43.54	-42.21
26	-36.40	-35.20	-38.80	-37.67	-36.53
27	-21.80	-23.43	-24.46	-24.18	-22.76
28	-36.30	-35.98	-38.73	-38.16	-36.29
29	-28.30	-26.17	-29.96	-28.85	-25.90
30	-14.00	-10.94	-16.83	-15.61	-16.75
31	-29.90	-26.69	-30.65	-29.66	-26.25
32	-1.80	-2.08	-0.33	-1.35	-1.24
33	-10.70	-7.84	-12.15	-13.05	-16.89
34	-25.60	-24.61	-25.38	-26.75	-28.36
35	-30.90	-29.92	-30.81	-30.82	-32.26
36	-39.80	-36.88	-36.66	-34.33	-36.76
37	-14.00	-16.31	-18.12	-16.12	-20.55
38	-64.40	-62.55	-66.55	-64.51	-59.37
39	-63.90	-64.19	-60.70	-64.00	-59.75
40	-65.80	-67.12	-66.39	-66.01	-66.53
41	-3.20	-2.21	-3.16	-4.30	-4.34

		LH20t-D4	LH24n-D4	ωLH25tdE-D4	LH25nP
MAE		2.25	3.65	2.47	4.86
RMSD		3.44	5.01	3.47	6.84
MSE		1.47	1.75	1.56	2.50
MaxE(−)		-5.66	-13.62	-2.60	-23.10
MaxE(+)		13.18	13.37	12.03	19.16
STDEV		3.11	4.68	3.09	6.36

Table S6. Comparison of ROST61 reaction-energy results and statistical data in kcal/mol for selected functionals.

	Ref.	LH20t-D4[S8]	LH24n-D4[S9]	ωLH25tdE-D4	LH25nP-D4
1	-40.05	-29.52	-30.34	-28.12	-26.47
2	-37.71	-46.09	-48.55	-47.49	-50.51
3	-12.59	-14.13	-14.21	-12.27	-13.83
4	-17.04	-16.33	-12.78	-15.01	-10.48
5	-10.66	-10.28	-11.92	-10.50	-12.69
6	-12.66	-8.93	-7.27	-7.83	-0.43
7	-14.73	-14.74	-14.76	-14.86	-13.50
8	-10.51	-9.08	-10.91	-9.61	-11.25
9	-12.76	-13.62	-13.11	-11.52	-10.57
10	-21.20	-26.01	-25.62	-24.23	-28.16
11	-8.44	-12.39	-12.51	-12.70	-17.59
12	-2.79	-1.99	-1.19	-1.65	-0.89
13	-178.96	-184.56	-198.16	-188.51	-196.83
14	-42.33	-43.84	-45.75	-44.07	-48.76
15	-7.17	-8.61	-7.91	-9.17	-7.98
16	-193.43	-191.40	-183.49	-185.98	-179.25
17	-200.10	-201.05	-193.56	-193.39	-190.12
18	-203.09	-202.72	-196.49	-196.59	-192.23
19	-4.94	1.98	6.50	0.60	14.96
20	-27.02	-30.24	-28.75	-28.39	-26.77
21	-25.09	-26.29	-29.42	-28.06	-25.80
22	-46.70	-47.76	-47.54	-45.60	-45.67
23	-115.35	-108.01	-109.42	-109.58	-112.16
24	-46.37	-45.16	-48.61	-46.30	-47.79
25	-19.39	-14.85	-11.87	-14.81	-3.59
26	-47.73	-50.03	-49.55	-49.12	-49.87
27	-25.86	-23.06	-23.58	-24.54	-22.09
28	-28.77	-25.91	-26.75	-27.20	-25.37
29	-11.13	-11.89	-11.77	-12.11	-12.25
30	-141.32	-141.58	-142.18	-142.27	-143.83
31	-63.73	-62.74	-58.11	-58.58	-53.52
32	-49.75	-50.14	-49.74	-51.14	-52.37
33	-10.64	-19.14	-20.47	-21.63	-26.42
34	-0.65	-0.81	-0.67	-0.49	-0.43
35	-5.46	-14.40	-15.12	-15.64	-19.50
36	-49.04	-48.18	-42.27	-48.54	-38.08
37	-40.62	-40.39	-39.66	-40.01	-36.12
38	-27.70	-28.56	-37.73	-27.53	-41.71
39	-44.99	-46.19	-49.02	-46.46	-48.64
40	-24.51	-25.65	-23.00	-23.53	-23.52
41	-5.96	-6.68	-6.46	-6.58	-5.99
42	-26.96	-26.69	-25.54	-23.28	-26.51
43	-80.58	-85.88	-85.07	-84.62	-82.11
44	-66.15	-71.15	-70.43	-69.75	-68.07
45	-42.83	-45.31	-47.56	-44.53	-43.90
46	-33.27	-32.91	-31.09	-29.29	-29.27
47	-58.47	-58.55	-55.91	-54.44	-54.00
48	-4.24	-3.74	-8.72	-5.70	-7.60
49	-35.26	-34.10	-34.27	-34.67	-32.77
50	-2.74	-3.68	-0.50	-1.12	-0.83
51	-29.77	-30.99	-34.39	-32.33	-32.25
52	-42.72	-46.54	-49.55	-43.08	-48.74
53	-30.78	-29.59	-30.34	-29.67	-28.83
54	-12.33	-12.79	-7.89	-11.84	-4.45
55	-4.61	-4.56	-4.59	-4.83	-5.16
56	-13.65	-13.92	-14.00	-14.03	-13.36
57	-31.89	-32.28	-28.45	-30.02	-23.88
58	-39.30	-40.76	-38.00	-38.38	-31.95
59	-29.89	-31.90	-30.12	-25.96	-24.43
60	-66.00	-65.31	-61.53	-61.52	-59.99
61	-69.48	-70.11	-71.11	-69.96	-71.20

		LH20t-D4[S8]	LH24n-D4[S9]	ωLH25tdE-D4	LH25nP-D4
MAE		2.23	3.87	2.87	5.44
RMSD		3.35	5.35	4.11	7.50
MSE		-0.52	-0.21	0.32	1.02
MaxE(−)		-8.94	-19.20	-10.99	-17.87
MaxE(+)		10.53	11.44	11.93	19.90
STDEV		3.34	5.39	4.13	7.50

Table S7. Comparison of MOBH28 reaction-barrier results in kcal/mol for selected functionals.

barrier	ref	LH20t-D4	LH24n-D4	ωLH25tdE-D4	LH25nP-D4
1_fwd	26.20	26.49	28.37	27.55	27.17
2_fwd	5.71	6.71	6.93	6.83	6.37
3_fwd	0.92	0.90	1.18	1.16	1.31
4_fwd	1.36	0.63	0.91	1.11	1.59
5_fwd	4.63	5.87	5.70	4.83	5.05
6_fwd	15.76	15.14	15.51	15.31	14.37
7_fwd	27.59	26.51	27.33	27.74	25.90
8_fwd	34.57	33.31	31.27	33.89	30.89
10_fwd	-4.29	0.86	-1.42	-0.82	1.88
11_fwd	29.49	29.05	27.28	24.83	30.39
12_fwd	5.50	4.86	4.65	4.68	6.07
13_fwd	20.65	18.34	23.48	19.08	21.93
14_fwd	10.10	10.65	10.30	10.11	11.13
15_fwd	20.66	17.95	20.24	20.24	18.50
16_fwd	35.45	34.98	33.14	34.18	30.86
21_fwd	8.41	8.58	9.79	11.00	10.75
22_fwd	13.84	12.35	14.71	15.11	14.63
23_fwd	29.45	28.53	31.97	31.58	29.05
26_fwd	25.83	20.80	22.03	16.59	18.67
27_fwd	14.05	12.82	12.63	13.11	11.58
28_fwd	30.18	31.04	29.52	24.11	26.58
29_fwd	14.72	15.52	14.19	14.60	15.38
30_fwd	9.79	11.58	10.64	9.75	10.26
31_fwd	2.91	7.65	6.68	3.38	5.99
32_fwd	20.18	20.90	22.14	21.38	20.90
33_fwd	1.05	0.01	1.14	3.60	3.20
34_fwd	29.16	27.63	26.57	28.85	26.38
35_fwd	17.28	16.09	15.84	16.90	15.72
1_rev	14.02	14.70	18.05	18.11	16.71
2_rev	22.25	20.00	23.39	22.62	24.42
3_rev	26.92	32.83	32.00	31.07	33.00
4_rev	8.25	11.71	12.13	12.08	14.31
5_rev	22.60	22.01	21.85	24.82	20.06
6_rev	14.61	12.95	12.57	12.63	10.38
7_rev	18.58	16.35	18.42	18.90	15.36
8_rev	31.82	29.34	23.63	29.53	18.39
10_rev	8.22	2.40	6.40	6.70	7.37
11_rev	82.34	80.96	84.56	84.65	84.28
12_rev	37.18	40.16	38.19	38.83	36.95
13_rev	47.99	48.83	52.70	48.70	44.67
14_rev	14.37	13.83	15.26	14.87	13.82
15_rev	74.98	81.15	78.83	79.49	74.47
16_rev	53.77	57.41	57.12	55.40	56.95
21_rev	8.41	8.58	9.79	11.00	10.75
22_rev	27.01	25.09	28.58	29.36	29.00
23_rev	20.35	20.47	22.58	22.56	22.30
26_rev	0.11	0.07	0.14	-0.13	-0.02
27_rev	2.29	1.97	1.81	2.89	2.32
28_rev	15.52	15.93	15.87	16.37	17.09
29_rev	31.19	29.88	29.60	30.66	29.41
30_rev	16.60	17.91	19.39	18.79	19.19
31_rev	12.90	11.72	11.85	12.11	12.49
32_rev	62.62	69.39	63.83	63.19	62.39
33_rev	7.86	10.32	11.43	7.05	11.91
34_rev	3.04	4.88	5.39	3.98	6.30
35_rev	-2.44	-2.30	-2.08	-2.05	-2.16

Table S8. Comparison of MOBH28 barrier-set statistical data in kcal/mol for selected functionals.

		LH20t-D4	LH24n-D4	ωLH25tdE-D4	LH25nP-D4
all	MAE	1.76	1.87	1.63	2.25
	RMSD	2.45	2.42	2.35	3.19
	MSE	0.17	0.56	0.36	0.00
	MaxE(−)	-5.82	-8.19	-9.24	-13.43
	MaxE(+)	6.77	5.08	4.51	6.17
	STDEV	2.47	2.38	2.35	3.22
only ΔE_{fwd}	MAE	1.43	1.52	1.57	1.94
	RMSD	1.97	1.89	2.58	2.60
	MSE	-0.19	0.06	-0.37	-0.31
	MaxE(−)	-5.03	-3.80	-9.24	-7.16
	MaxE(+)	5.15	3.77	3.47	6.17
	STDEV	2.00	1.92	2.60	2.63
only ΔE_{rev}	MAE	2.09	2.22	1.68	2.56
	RMSD	2.85	2.86	2.10	3.68
	MSE	0.54	1.07	1.10	0.31
	MaxE(−)	-5.82	-8.19	-2.29	-13.43
	MaxE(+)	6.77	5.08	4.51	6.08
	STDEV	2.85	2.70	1.82	3.74

Table S9. Statistical analysis of selected optimized bond lengths for the LMGB35 test set with selected functionals. All results include D4 corrections. "scLH23t" refers to the scLH23t-mBR-P functional. Calculations were performed with def2-QZVPPD basis sets and gridsize 3.

	Ref.	LH20t	PBE	PBE0	scLH22ta	scLH22t	scLH23t	LH24n	LH25nP
H_2 (H-H)	74.50	73.40	74.97	74.43	73.54	73.34	73.47	73.56	74.42
HF (H-F)	91.80	91.61	93.01	91.77	91.64	91.93	91.61	91.17	89.79
H_2O (H-O)	95.70	95.40	96.88	95.74	95.45	95.54	95.41	95.92	95.48
HOF (O-H)	96.60	96.33	97.96	96.64	96.40	96.63	96.35	96.71	95.38
OH (O-H)	97.00	96.69	98.29	97.03	96.75	96.74	96.71	96.97	96.64
NH_3 (N-H)	101.10	100.63	102.10	101.13	100.71	100.57	100.65	101.24	102.03
OH^+ (O-H)	103.20	103.08	104.73	103.16	103.18	103.06	103.13	102.58	102.28
NH (N-H)	103.70	103.11	104.93	103.72	103.23	102.91	103.18	103.74	104.78
C_2H_2 (C-H)	106.40	106.12	107.00	106.37	106.18	106.21	106.13	106.16	107.19
NO^+ (N-O)	105.30	104.99	106.90	105.29	105.05	105.50	105.00	105.33	104.49
HCN (H-C)	106.80	106.48	107.48	106.76	106.55	106.57	106.50	106.51	107.59
NH^+ (N-H)	107.60	106.89	109.07	107.56	107.05	106.84	106.99	107.18	107.44
C_2H_4 (C-H)	108.30	107.85	109.07	108.34	107.94	107.80	107.88	108.10	108.99
CH_4 (CH)	108.80	108.40	109.52	108.83	108.49	108.23	108.43	108.69	109.29
N_2 (N-N)	108.90	108.56	110.20	108.87	108.59	108.92	108.57	108.90	108.22
CH_2O (O-H)	110.70	109.76	111.72	110.72	109.93	109.78	109.84	110.14	111.47
N_2^+ (N-N)	110.10	109.93	111.38	110.14	110.30	110.46	110.20	110.28	110.01
O_2^+ (O-O)	109.80	109.50	112.12	109.78	109.57	110.05	109.52	110.15	108.39
CH (C-H)	112.40	111.11	113.58	112.38	111.29	111.00	111.22	111.63	112.91
CO (C-O)	112.20	112.00	113.54	112.22	112.03	112.41	112.00	112.24	111.87
HCN (C-N)	114.50	114.04	115.74	114.46	114.09	114.41	114.06	114.34	114.84
CO_2 (C-O)	115.60	115.35	117.05	115.60	115.39	115.78	115.36	115.78	115.61
C_2H_2 (C-C)	119.60	119.14	120.65	119.58	119.16	119.38	119.15	119.32	119.65
CH_2O (C-O)	119.50	119.66	120.77	119.48	119.69	120.03	119.66	120.11	119.76
BO (B-O)	119.90	119.59	121.27	119.88	119.65	120.05	119.60	120.10	119.80
O_2 (O-O)	119.20	119.05	121.77	119.18	119.06	119.18	119.05	119.80	118.55
BH (B-H)	124.00	121.71	125.04	123.94	121.95	121.66	121.82	122.66	123.74
BF (B-F)	125.90	126.04	127.24	125.90	126.06	126.59	126.04	125.75	125.54
CF (C-F)	126.70	126.89	128.54	126.67	126.89	127.72	126.89	126.85	125.24
NF (N-F)	130.30	130.61	132.71	130.33	130.62	131.52	130.62	131.97	129.56
F_2^+ (F-F)	127.20	127.04	131.56	127.26	127.15	128.75	127.05	126.66	124.52
C_2H_4 (C-C)	132.20	132.31	133.22	132.24	132.32	132.33	132.33	132.59	132.60
F_2 (F-F)	137.50	137.62	141.32	137.53	137.84	139.99	137.66	138.28	135.43
HOF (O-F)	140.50	140.71	144.48	140.58	140.79	142.68	140.73	142.42	140.03
B_2 (B-B)	161.30	161.62	158.50	161.33	160.65	160.06	160.13	162.16	162.37

	LH20t	PBE	PBE0	scLH22ta	scLH22t	scLH23t	LH24n	LH25nP
MAE	0.42	1.57	0.03	0.38	0.65	0.42	0.45	0.72
RMSE	0.59	1.82	0.04	0.53	0.93	0.59	0.64	0.94
MSE	-0.33	1.41	0.00	-0.27	-0.01	-0.34	0.03	-0.25
MaxE(−)	-2.29	-2.80	-0.07	-2.05	-2.34	-2.18	-1.34	-2.68
MaxE(+)	0.32	4.36	0.08	0.34	2.49	0.32	1.92	1.08
STDEV	0.50	1.17	0.04	0.46	0.95	0.49	0.65	0.92

Table S10. MAEs for the LMGB35 test set in pm using the def2-TZVP basis set used in Ref. S10. Results for DM21 obtained with numerical gradients.

	MAE [pm]
PBE0-D3	0.14
B3LYP-D3 (this paper)	0.60
LH25nP-D4	0.68
LH24n-D4	0.47
Skala	1.40
B3LYP-D3 (Skala paper)	0.70
PBE0-D3 (DM21 geometry paper)	0.95
DM21	0.62

.

Table S11. Optimized metal-ligand bond lengths for the TMC32 test set and statistical analysis with selected functionals. All results include D4 corrections. “scLH23t” refers to the scLH23t-mBR-P functional. Results obtained with the x2c-TZVPall basis set and the 1c-X2C scalar-relativistic method

	Ref	LH20t	PBE	PBE0	scLH22ta	scLH22t	scLH23t	LH24n	LH25nP
$Sc(acac)_3$ (Sc-O)	207.60	209.81	210.29	208.77	211.39	209.88	209.63	211.08	210.16
$TiCl_4$ (Ti-Cl)	216.90	215.71	217.92	216.08	216.06	215.72	215.71	216.95	216.94
$Ti(CH_3)Cl_3$ (Ti-C)	204.70	202.61	204.24	202.06	203.52	202.58	202.59	203.03	202.45
$Ti(CH_3)Cl_3$ (Ti-Cl)	218.50	217.32	218.93	217.55	217.42	217.37	217.34	218.76	218.27
$Ti(CH_3)_2Cl_2$ (Ti-C)	205.80	203.78	205.15	203.29	204.45	203.73	203.73	204.37	203.74
$Ti(CH_3)_2Cl_2$ (Ti-Cl)	219.60	219.09	220.09	219.14	219.06	219.18	219.12	220.55	220.56
$Ti(BD_4)_3$ (Ti-B)	217.50	214.06	214.30	213.98	213.59	220.82	214.09	215.97	215.27
$Ti(BD_4)_3$ (Ti-D^{br})	198.40	192.53	193.58	193.35	192.43	197.70	192.76	195.00	194.89
VOF_3 (V=O)	157.00	153.49	157.18	153.99	155.43	153.88	153.57	153.81	153.06
VOF_3 (V-F)	172.90	171.08	173.13	171.00	172.46	171.15	171.08	171.75	170.42
VF_5 (V-Fax)	173.40	173.06	176.14	173.27	174.54	173.09	173.06	173.51	171.56
VF_5 (V-Feq)	170.80	169.34	172.14	169.36	171.01	169.39	169.35	169.57	168.70
$VOCl_3$ (V=O)	157.30	153.43	156.98	153.75	155.63	153.92	153.53	153.72	153.34
$VOCl_3$ (V-Cl)	213.80	212.15	214.47	212.54	212.69	212.22	212.16	213.32	213.48
$V(N(CH_3)_2)_4$ (V-N)	187.90	194.32	194.30	193.73	195.61	194.47	194.60	196.22	196.13
$V(Cp)(CO)_4$ (V-C^{CO})	196.30	192.48	192.31	192.04	192.69	192.51	192.46	195.53	196.06
CrO_2F_2 (Cr=O)	157.40	152.78	156.57	153.26	155.21	153.46	152.93	153.05	151.70
CrO_2F_2 (Cr-F)	171.90	169.67	171.79	169.60	171.38	169.91	169.71	170.48	169.03
CrO_2Cl_2 (Cr=O)	157.70	153.06	156.75	153.42	155.62	153.84	153.24	153.32	151.91
CrO_2Cl_2 (Cr-Cl)	212.20	209.71	211.93	210.06	210.35	209.90	209.74	210.89	210.63
$CrO_2(NO_3)_2$ (Cr=O)	158.40	153.05	156.95	153.47	155.76	153.85	153.25	153.28	152.05
$CrO_2(NO_3)_2$ (Cr-O)	195.40	189.61	192.57	190.36	191.08	189.68	189.61	191.56	192.75
$Cr(C_6H_6)_2$ (Cr-C)	215.00	211.99	213.41	212.64	210.77	211.99	212.01	215.63	216.49
$Cr(C_6H_6)(CO)_3$ (Cr-C^{Ar})	220.80	217.98	220.78	218.92	217.14	218.02	217.99	221.98	220.98
$Cr(C_6H_6)(CO)_3$ (Cr-C^{CO})	186.30	183.53	183.38	183.24	183.58	183.53	183.54	186.21	187.20
$Cr(NO)_4$ (Cr-N)	175.00	170.80	173.49	170.97	173.12	171.52	171.21	171.68	171.54
MnO_3F (Mn=O)	158.60	153.26	157.18	153.74	156.25	154.51	153.60	153.37	152.29
MnO_3F (Mn-F)	172.40	169.85	171.50	169.58	171.49	170.28	170.04	170.86	169.08
$MnCp(CO)_3$ (Mn-C^{Cp})	214.70	211.97	214.60	213.23	210.34	212.00	212.00	217.73	221.80
$MnCp(CO)_3$ (Mn-C^{CO})	180.60	177.98	177.83	177.64	177.84	177.99	177.98	182.15	186.22
$Fe(CO)_5$ (Fe-C)^{mean}	182.90	179.01	179.45	178.53	179.26	179.01	179.01	181.82	183.44
$Fe(CO)_3$(tmm) (Fe-C^{CO})	181.00	177.51	177.16	176.96	177.29	177.49	177.50	182.53	193.08
$Fe(CO)_3$(tmm) (Fe-C^{cent})	193.80	191.75	194.49	192.02	192.14	191.90	191.95	193.42	196.48
$Fe(CO)_3$(tmm) (Fe-C^{CH2})	212.30	208.77	212.91	209.54	208.63	208.68	208.64	211.81	216.35
$Fe(CO)_2(NO)_2$ (Fe-C)^{mean}	187.20	180.87	180.42	179.72	181.13	180.68	180.75	182.90	189.09
$Fe(CO)_2(NO)_2$ (Fe-N)	167.40	163.21	166.39	163.48	165.37	163.73	163.44	164.11	170.12
$FeCp_2$ (Fe-C)	206.40	202.94	203.51	203.50	201.07	202.94	202.95	210.83	216.85
$Fe(C_2H_4)(CO)_4$ (Fe-C^{et})	211.70	209.05	216.66	209.10	209.71	209.07	209.08	216.03	212.65
$Fe(C_2H_4)(CO)_4$ (Fe-C^{ax})	181.50	179.64	179.27	179.26	179.07	179.60	179.64	185.14	196.70
$Fe(C_2H_4)(CO)_4$ (Fe-C^{eq})	180.60	177.71	177.88	177.25	177.88	177.71	177.71	180.56	191.78
$Fe(C_5(CH_3)_5)(P_5)$ (Fe-P)	237.70	233.71	235.81	235.27	230.94	233.75	233.77	241.15	252.20
$CoH(CO)_4$ (Co-Ceq)	181.80	179.11	178.43	178.15	178.62	178.74	178.71	184.78	186.10
$Co(CO)_3(NO)$ (Co-N)	165.80	161.98	165.29	162.23	164.00	162.45	162.18	162.99	162.62
$Co(CO)_3(NO)$ (Co-C)	183.00	179.77	179.23	178.72	179.68	179.66	179.73	183.36	184.78
$Ni(CO)_4$ (Ni-C)	182.50	181.35	180.72	180.36	181.25	181.35	181.35	185.07	187.80
$Ni(acac)_2$ (Ni-O)	187.60	184.01	184.58	183.60	185.19	184.05	184.02	188.53	189.66
$Ni(PF_3)_4$ (Ni-P)	209.90	207.86	209.06	207.42	207.71	207.42	207.87	210.94	213.56
$CuCH_3$ (Cu-C)	188.40	188.41	186.98	187.47	188.53	188.31	188.36	193.73	198.44
CuCN (Cu-C)	183.20	182.08	179.20	181.29	182.39	182.07	182.09	188.48	192.35
$Cu(acac)_2$ (Cu-O)	191.40	191.67	193.45	191.18	192.99	191.71	191.67	194.92	194.94

	LH20t	PBE	PBE0	scLH22ta	scLH22t	scLH23t	LH24n	LH25nP
MAE	2.98	1.98	2.92	2.59	2.75	2.94	2.41	4.19
RMSE	3.35	2.57	3.28	3.10	3.10	3.31	3.02	5.57
MSE	-2.62	-1.00	-2.64	-2.00	-2.25	-2.58	-0.05	1.54
MaxE(−)	-6.33	-6.78	-7.48	-6.76	-6.52	-6.45	-5.23	-6.35
MaxE(+)	6.42	6.40	5.83	7.71	6.57	6.70	8.32	15.20
STDEV	2.11	2.39	1.97	2.39	2.15	2.10	3.05	5.41

References

[S1] D. Hendrycks and K. Gimpel, arXiv p. DOI:10.48550/arXiv.1606.08415 (2016).

[S2] D. E. Rumelhart, G. E. Hinton, and R. J. Williams, Nature **323**, 533 (1986).

[S3] J. Kirkpatrick, B. McMorrow, D. H. P. Turban, A. L. Gaunt, J. S. Spencer, A. G. D. G. Matthews, A. Obika, L. Thiry, M. Fortunato, D. Pfau, et al., Science **374**, 1385 (2021).

[S4] A. Patra, S. Jana, H. Myneni, and P. Samal, Phys. Chem. Chem. Phys. **21**, 19639 (2019).

[S5] J. P. Perdew, K. Burke, and M. Ernzerhof, Phys. Rev. Lett. **77**, 3865 (1996).

[S6] J. P. Perdew and Y. Wang, Phys. Rev. B **45**, 13244 (1992).

[S7] A. Wodyński, A. V. Arbuznikov, and M. Kaupp, J. Chem. Phys. **158**, 244117 (2023).

[S8] R. Grotjahn and M. Kaupp, Isr. J. Chem. **63**, e202200021 (2022).

[S9] A. Wodyński and M. Kaupp, J. Chem. Theory Comput. **21**, 7419 (2025).

[S10] G. Luise, C.-W. Huang, T. Vogels, D. P. Kooi, S. Ehlert, S. Lanius, K. J. H. Giesbertz, A. Karton, D. Gunceler, M. Stanley, et al., *Accurate and scalable exchange-correlation with deep learning* (2025), 2506.14665, URL https://arxiv.org/abs/2506.14665.
